# Supplementary material for: Ultrasound-guided puncture drainage versus surgical incision drainage for deep neck space abscesses: a protocol for a systematic review with meta-analysis and trial sequential analysis
Source: BMJ Open. 2024 Jan 4;14(1):e077631. doi: 10.1136/bmjopen-2023-077631 (PMC10773404; doi:10.1136/bmjopen-2023-077631)
Supplement: Supplementary data [file bmjopen-2023-077631supp001.pdf]

Supplementary Appendix file 1: Search strategy

Search strategy of PubMed as follows:

#1 "deep"[All Fields] AND ("neck"[MeSH Terms] OR "neck"[All Fields]) AND ("space"[All Fields] OR "space s"[All Fields] OR "spaces"[All Fields])) OR "deep neck space"[Title/Abstract] "parapharyngeal space"[Title/Abstract] OR "submandibular space"[Title/Abstract] OR "masseter space"[Title/Abstract] OR "retropharyngeal space "[Title/Abstract] OR "sublingual space "[Title/Abstract] OR "prevertebral space "[Title/Abstract] OR "carotid space"[Title/Abstract]

#2 "infections"[MeSH Terms] OR "infection" [Text Word] OR "infections" [Text Word] OR "infections"[Title/Abstract] OR "infection"[Title/Abstract]

#3 "abscess"[MeSH Terms] OR "abscess " [Text Word] OR "abscesses" [Text Word] OR "abscess " [Title/Abstract] OR "abscesses "[Title/Abstract]

#4 #2 OR #3

#5 #1 and #4

#6 "Ultrasonography, Interventional"[Mesh] OR "Ultrasonics"[Mesh] OR "Ultrasonography"[Mesh] OR "ultrasound-guided "[Title/Abstract]

#7 "Surgical Procedures, Operative"[Mesh] OR ("Surgical"[All Fields] AND "procedures"[All Fields] AND "operative"[All Fields]) OR "operative surgical procedures"[All Fields] OR "Surgical"[All Fields] OR "surgically"[All Fields] OR "surgicals"[All Fields]) AND "Surgical"[Title/Abstract]

#8 "Randomized Controlled Trial" [Publication Type] OR "Randomized Controlled Trials as Topic"[Mesh] OR "Randomized Controlled Trial, Veterinary" [Publication Type] OR "Controlled Clinical Trial" [Publication Type] OR “randomized” [Title/Abstract] OR “randomized” [Title/Abstract] OR “Placebo” [Title/Abstract] OR “randomly” [Title/Abstract] OR “Clinical trial” [Title]

#9 #5 and #6 and #7 and #8

Search strategy of Cochrane library as follows:

#1 (deep neck space or parapharyngeal space or submandibular space or masseter space or retropharyngeal space or sublingual space or prevertebral space or carotid space): ti,ab,kw

#2 MeSH descriptor: Abscess Explode all trees

#3 (abscess): ti,ab,kw

#4 #2or #3

#5 MeSH descriptor: Infections Explode all trees

#6 (infections): ti,ab,kw

#7 #5or #6

#8 #4or #7

#9 #1 and #8

#10 MeSH descriptor: Ultrasonography Explode all trees

#11 (ultrasound-guided): ti,ab,kw

#12 #10 or #11

#13MeSH descriptor: Surgical Procedures, Operative Explode all trees

#14 (surgical): ti,ab,kw

#15 #13or #14

#16 (controlled clinical trial):pt or (randomized controlled trial):pt or (random*): ti,ab,kw or (Clinical trial):ti,ab,kw

#17 #9 and #12 and #15 and #16

Search strategy of Web of Science as follows:

#1 TS= (deep neck space or parapharyngeal space or submandibular space or masseter space or retropharyngeal space or sublingual space or prevertebral space or carotid space)

#2 TS= (infection)

#3 TS= (abscess)

#4 #2 or #3

#5 #1and #4

#6 TS= (surgical)

#7 TS= (ultrasound)

#8 #6 and #7

#9 TS= (random* or Clinical trial)

#10 #5 and #8 and #9

Search strategy for Ovid Medline as follows:

#1(deep neck space or parapharyngeal space or submandibular space or masseter space or retropharyngeal space or sublingual space or prevertebral space or carotid space).mp.

#2 exp infections/

#3 (infections).mp.

#4 #2 or # 3

#5 exp abscess /

#6 (abscess).mp.

#7 #5 or #6

#8 #4 or #7

#9 #1 and #8

#10 exp Surgical/

#11 (Surgical).mp.

#12 #10 or #11

#13 exp ultrasound/

#14 (ultrasound).mp.

#15 #13 or #14

#16 #12 and #15

#17 randomized controlled trial.pt.

#18 controlled clinical trial.pt.

#19 randomized.ab.

#20 placebo.ab.

#21 clinical trials as topic.sh.

#22 randomly.ab.

#23 trial.ti.

#24 #17 or #18 or #19 or #20 or #21 or #22 or #23

#25 #9 and #16 and #24

Search strategy for Embase as follows:

#1 (deep neck space or parapharyngeal space or submandibular space or masseter space or retropharyngeal space or sublingual space or prevertebral space or carotid space) .mp.

#2 exp Abscess/

#3 (abscess).mp.

#4 #2 or # 3

#5 exp infections /

#6 (infections).mp.

#7 #5 or # 6

#8 #4 or # 7

#9 #1 and #8

#10 exp ultrasonography /

#11 (ultrasound-guided).mp.

#12 #10 or # 11

#13 #9 and #12

#14 exp Surgical Procedures /

#15 (Surgical).mp.

#16 #14 or # 15

#17 exp randomized controlled trial/

#18 (random*).mp.

#19 (placebo*).mp.

#20 Clinical trial.mp.

#21 clinical trials as topic.sh.

#22 #17 or #18 or #19 or #20 or #21

#23 #13 and #16 and #22

WHO ICTRP Trial registry

http://apps.who.int/trialsearch (WHO ICTRP register) will be searched via the advanced search page. Search terms were: (deep neck space OR parapharyngeal space OR submandibular space OR masseter space OR retropharyngeal space OR sublingual space OR prevertebral space OR carotid space) AND (abscess OR infections) AND (ultrasound).

Clinicaltrials.gov search strategy

http://clinicaltrials.gov (NIH register) will be searched via advanced search page. Search terms were: Condition or disease: (deep neck space OR parapharyngeal space OR submandibular space OR masseter space OR retropharyngeal space OR sublingual space OR prevertebral space OR carotid space) AND (abscess OR infections);

Study type: Interventional Study (clinical trial);

Intervention/treatment: (ultrasound-guided);

Chinese Clinical Trial Registry

https://www.chictr.org.cn/index.html (Chinese Clinical Trial Registry, ChiCTR) will be searched via Subject of Registration. Search terms were: 颈部脓肿；下颌脓肿；扁桃体周围脓肿；咽旁间隙脓肿；颌下间隙脓肿；咬肌间隙脓肿；咽后间隙脓肿；舌下间隙脓肿；椎前间隙脓肿；颈动脉间隙脓肿；颈部感染；下颌感染；扁桃体周围感染；咽旁间隙感染；颌下间隙感染；咬肌间隙感染；咽后间隙感染；舌下间隙感染；椎前间隙感染；颈动脉间隙感染；

Chinese database

China National Knowledge Infrastructure (CNKI) search strategy

(颈部[全部字段]or 下颌[全部字段]or 扁桃[全部字段]or 咽旁[全部字段]or 颌下[全部字段]or 咬肌[全部字段]or 咽后[全部字段]or 舌下[全部字段]or 椎前[全部字段]or 颈动脉 [全部字段]) and (脓肿[全部字段] or 感染[全部字段]) and (超声[全部字段] or B 超[全部字段]) and (随机[全部字段] or 对照[全部字段])

Chinese BioMedical Literature (CBM)

(颈部[全部字段]or 下颌[全部字段]or 扁桃[全部字段]or 咽旁[全部字段]or 颌下[全部字段]or 咬肌[全部字段]or 咽后[全部字段]or 舌下[全部字段]or 椎前[全部字段]or 颈动脉 [全部字段]) and (脓肿[全部字段] or 感染[全部字段]) and (超声[全部字段] or B 超[全部字段]) and (随机[全部字段] or 对照[全部字段])

VIP database

关键词=(颈部 or 下颌 or 扁桃 or 咽旁 or 颌下 or 咬肌 or 咽后 or 舌下 or 椎前 or 颈动脉) AND 关键词= (脓肿 or 感染) AND 关键词= (超声 or B 超) AND 关键词= (随机 or 对照)

Wan fang database.

(颈部[全部字段]or 下颌[全部字段]or 扁桃[全部字段]or 咽旁[全部字段]or 颌下[全部字段]or 咬肌[全部字段]or 咽后[全部字段]or 舌下[全部字段]or 椎前[全部字段]or 颈动脉 [全部字段]) and (脓肿[全部字段] or 感染[全部字段]) and (超声[全部字段] or B 超[全部字段]) and (随机[全部字段] or 对照[全部字段])
