# Supplementary material for: Ultrasound-guided puncture drainage versus surgical incision drainage for deep neck space abscesses: a protocol for a systematic review with meta-analysis and trial sequential analysis
Source: BMJ Open. 2024 Jan 4;14(1):e077631. doi: 10.1136/bmjopen-2023-077631 (PMC10773404; doi:10.1136/bmjopen-2023-077631)
Supplement: Author's manuscript [file bmjopen-2023-077631.draft_revisions.pdf]

BMJ Open is committed to open peer review. As part of this commitment we make the peer review history of every article we publish publicly available.

When an article is published we post the peer reviewers' comments and the authors' responses online. We also post the versions of the paper that were used during peer review. These are the versions that the peer review comments apply to.

The versions of the paper that follow are the versions that were submitted during the peer review process. They are not the versions of record or the final published versions. They should not be cited or distributed as the published version of this manuscript.

BMJ Open is an open access journal and the full, final, typeset and author-corrected version of record of the manuscript is available on our site with no access controls, subscription charges or pay-per-view fees (http://bmjopen.bmj.com).

If you have any questions on BMJ Open's open peer review process please email info.bmjopen@bmj.com

BMJ Open

Ultrasound-guided puncture drainage vs surgical incision drainage for deep neck space abscesses: a protocol for a systematic review with meta-analysis and trial sequential analysis

Journal:	*BMJ Open*
Manuscript ID	bmjopen-2023-077631
Article Type:	Protocol
Date Submitted by the Author:	10-Jul-2023
Complete List of Authors:	Nie, Wei; Sichuan University West China Hospital, Department of Anesthesiology<br>Du, Li; Sichuan Cancer Hospital and Research Institute, Department of Anesthesiology<br>Chen, Guo; Sichuan University West China Hospital, Department of Anesthesiology<br>Zheng, Yongbo; Sichuan University West China Hospital, Department of Otorhinolaryngology, Head & Neck Surgery<br>Zhang, Weiyi; Sichuan University West China Hospital, Department of Anesthesiology<br>Zheng, Jianqiao; Sichuan University West China Hospital, Department of Anesthesiology
Keywords:	Ultrasound < RADIOLOGY & IMAGING, Randomized Controlled Trial, Head & neck surgery < OTOLARYNGOLOGY

SCHOLARONE™
Manuscripts

TITLE PAGE

Ultrasound-guided puncture drainage vs surgical incision drainage for deep neck space abscesses: a protocol for a systematic review with meta-analysis and trial sequential analysis

Authors

Wei Nie[1] E-mail: nieweione@163.com

Li Du[2] E-mail: huaying-duli@163.com

Guo Chen[1] E-mail: Anesthesiology_SCU@163.com

Yongbo Zheng[3] E-mail: zheng_yongbo@126.com

Weiyi Zhang [1] E-mail: zhangweiyi@wchscu.cn

*Jianqiao Zheng [1] E-mail: zhjq1983@163.com

[1] Department of Anesthesiology, West China Hospital, Sichuan University No. 37th, Guoxue Alley, Wuhou District, Chengdu, Sichuan, China.

[2] Department of Anesthesiology, Sichuan Cancer Hospital & Institute, Sichuan Cancer Center, School of Medicine, University of Electronic Science and Technology of China, No.55th, People's South Road, Chengdu, Sichuan, China.

[3] Department of Otorhinolaryngology, Head & Neck Surgery, West China Hospital, Sichuan University, No. 37th, Guoxue Alley, Wuhou District, Chengdu, Sichuan, China.

*Corresponding Author

Name: Jianqiao Zheng

E-mail: zhjq1983@163.com

Phone: +86-28-8542-3593

Address: Department of Anesthesiology, Sichuan University West China Hospital, No. 37th, Guoxue Alley, Wuhou District, Chengdu 610041, Sichuan, China.

Words: 3010

For peer review only

ABSTRACT

Introduction Deep neck space abscesses (DNAs) are serious surgical emergencies, associated with life-threatening complications. Surgical incision-and-drainage combined with antibiotics is the main treatment for DNAs, but drawbacks still exist. Ultrasound-guided puncture drainage is an alternative treatment for some DNAs with limited clinical evidence. Hence, the optimal drainage technique for the treatment of DNAs remains unclear. Therefore, we will perform a protocol for a systematic review and meta-analysis to identify the efficacy of ultrasound-guided puncture drainage for DNAs.

Methods and analysis PubMed, Ovid Medline, Cochrane Library, Embase, Web of Science, China National Knowledge Infrastructure, Wanfang database, VIP database and trial registry databases will be searched from inception to September 2023 to identify randomized controlled trials of patients diagnosed DNAs accepting ultrasound-guided puncture drainage. Primary outcome will be the length of hospital stay. Secondary outcomes will be the cure rate, incidence of retreatment, complications and overall cost to the healthcare system. Fixed-effects or random-effects model will be used according to the statistical heterogeneity. Mean differences or standardized mean differences with 95% confidence intervals (CIs) for continuous data and risk ratio (RR) with 95% Cis for dichotomous data. The Cochrane risk-of-bias tool 2, Grading of

For peer review only - http://bmjopen.bmj.com/site/about/guidelines.xhtml

Recommendations Assessment, Development and Evaluation (GRADE) and trial sequential analysis will be conducted to evaluate the evidence quality and control the random errors. Funnel plots and Egger's regression test will be performed to evaluate publication bias.

Ethics and dissemination Ethical approval was not required for this systematic review protocol. The results will be disseminated through peer-reviewed publications.

Keywords deep neck space, abscesses, infection, ultrasound, surgical, meta-analysis, randomized controlled trial.

PROSPERO registration number CRD42023441031

Strengths and limitations of the study

► Application of Preferred Reporting Items for Systematic Review and Meta-Analysis Protocols (PRISMA-P) guidelines for better quality of meta-analytical results.

► Trial sequential analysis to control random errors of all outcomes.

► Funnel plots and Egger's regression test to assess the publication bias.

► Cochrane risk-of-bias tool 2 (ROB-2) to assess the risk of bias.

► GRADE to confirm the quality of the evidence.

► Data in the graphs will be extracted and data that cannot be analyzed will be transformed.

► Subgroup analysis based on participants' ages, and different locations of DNAs for heterogeneity assessment.

For peer review only - http://bmjopen.bmj.com/site/about/guidelines.xhtml

INTRODUCTION

Deep neck space abscesses (DNAs) are serious otolaryngology-head and neck surgery emergencies, defined as bacterial infections in the potential spaces and fascial planes of the neck, which could result in significant morbidity with potential mortality.[1-4] Dental infection is the most common cause of DNAs, and the prevalence of DNAs has been reduced with the advent of broad-spectrum antibiotics.[5] However, DNAs remains serious, spreads rapidly, and is associated with life-threatening complications.[6-9] Common and potentially life-threatening complications range from 15% to 35% in patients with DNAs[5,10,11], including airway compromise, descending mediastinitis, pericarditis, pneumonia, pleural empyema, carotid arterial erosion, meningitis, sepsis, respiratory distress, jugular vein thrombosis, disseminated intravascular coagulation, and extracranial or intracranial extension of infection.[5,8-13] Therefore, the early detection of abscesses and thorough treatment are crucial.

Traditionally, the main treatment method for DNAs is complete surgical incision-and-drainage with appropriate administration of antibiotics.[14-19] However, surgical drainage has several drawbacks. First, the risk of inadvertent injuries to neighboring vital structures (including the internal jugular vein, carotid artery and cranial nerves) is associated with devastating complications. Second, surgical trauma danger is increased as the inflammatory anatomy tissue is obscure and easily bled during incision

For peer review only - http://bmjopen.bmj.com/site/about/guidelines.xhtml

when the abscess is deep. Third, patients suffering from surgical trauma stress become frail and more sensitive to pathogenic bacteria in the environment, increasing the risk of infection-related complications. Fourth, tumor-related DNAs could cause tumor dissemination during open surgical incision-and-drainage. Fifth, surgical incision-and-drainage was performed under general anesthesia in most circumstances, which will increase the anesthesia-related risk, especially in poor medical condition DNAs patients. Finally, surgical incision scars increase the risk of incision disunion and cause aesthetic defects. Therefore, a noninvasive, effective alternative to open surgical incision drainage may be beneficial to DNAs patients.

Minimally invasive techniques, such as ultrasound-guided puncture drainage, have been used to treat abscesses of the head and neck, even some DNAs.[20-37] Ultrasound-guided puncture drainage can avoid the above drawbacks of surgical incision drainage, and provide several advantages. First, ultrasound-guided puncture drainage can accurately locate the DNAs, eliminating trauma to the neighboring vital structures, and avoiding tumor dissemination during surgical incision. Second, the drainage tube can be placed under the real-time guidance of ultrasound, which is beneficial for thoroughly draining the abscess. Third, the real-time capability of ultrasound could facilitate dynamic observation after drainage and monitoring the adequacy of drainage. Finally, ultrasound-guided puncture

For peer review only - http://bmjopen.bmj.com/site/about/guidelines.xhtml

drainage does not cause skin nonunion or aesthetic defects. Several studies have shown that ultrasound-guided puncture drainage is a more effective option than surgical incision drainage for some DNAs.[20-36] In addition, ultrasound-guided puncture drainage can reduce the length of hospital stay.[25-29] However, most of these studies were case series, reviews and retrospective studies, and prospective and randomized controlled trials are rare.[20-36] In this situation, controversies still exist in the selection of drainage technique for the treatment of DNAs.[37-38]

Therefore, it is necessary to conduct a systematic review and meta-analysis to analyze the clinical efficacy of ultrasound-guided puncture drainage for DNAs. The outcomes of this systematic review will provide evidence for better clinical decision making and possible future directions for further clinical trials.

Objectives

We are performing this systematic review protocol with meta-analysis and trial sequential analysis (TSA) of randomized clinical trials to evaluate the clinical efficacy and safety of ultrasound-guided puncture drainage for DNAs.

METHODS AND ANALYSIS

Design and registration of the review

We devised this protocol according to the Preferred Reporting Items for Systematic Reviews and Meta-Analyses Protocols (PRISMA-P)

For peer review only - http://bmjopen.bmj.com/site/about/guidelines.xhtml

guidelines that have been registered with PROSPERO 2023 (registration number: CRD42023441031).[39] We will perform this systematic review and meta-analysis based on the Cochrane Handbook and report the results following the PRISMA statement.[40,41] This study is anticipated to begin searching in September 2023 and will be completed in March 2024.

Inclusion criteria for study selection

Types of studies

Only randomized controlled trials (RCTs) involving the clinical efficacy of ultrasound-guided puncture drainage for DNAs will be included. There will be no language restrictions.

The exclusion criteria were as follows: (1) studies comparing ultrasound-guided needle puncture aspiration and ultrasound-guided catheter drainage or studies comparing ultrasound-guided needle puncture aspiration with other minimally invasive techniques (CT-guided needle puncture aspiration); (2) studies with data that could not be used for statistical analysis after data transformation, or studies with incomplete data, or original data that could not be obtained after contacting the corresponding authors; and (3) duplicate publications, letters or editorials, abstracts from conferences, and reviews.

Types of participants

Participants diagnosed DNAs who accept ultrasound-guided puncture drainage will be included. There will be no limitations on participants' age,

For peer review only - http://bmjopen.bmj.com/site/about/guidelines.xhtml

gender, ethnicity, body mass index (BMI) or American Society of Anesthesiologists (ASA) classification.

Types of interventions/controls

The intervention group will be the participants with DNAs accepting any kind of ultrasound-guided puncture drainage, while the control group will receive any kind of surgical incision drainage for DNAs.

Types of outcome measures

Primary outcomes

The primary outcome will be the length of hospital stay.

Secondary outcomes

1. The cure rate;
2. Incidence of retreatment (including secondary puncture drainage or incision drainage);
3. Incidence of complications: any kind of complications will be included. Complication criteria included drainage-related mechanical injury (vascular injury, nerve injury, airway injury, and bleeding), infectious complications (sepsis, mediastinal infection, pericarditis, pneumonia, arterial infection), airway obstruction, jugular vein thrombosis, skin nonunion and other puncture complications;
4. Overall cost to the healthcare system;

Search strategy

Two reviewers (NW and DL) will independently perform the search

For peer review only - http://bmjopen.bmj.com/site/about/guidelines.xhtml

For peer review only

and any disagreements will be arbitrated by a third reviewer (Z-WY). English and Chinese electronic databases will be searched from inception to September 2023 for published literature. English databases (including PubMed, Ovid Medline, Cochrane Library, Embase and Web of Science) and Chinese databases [China National Knowledge Infrastructure (CNKI), Wanfang database and VIP Database] will be searched. In addition, reference lists of each study and the trial registry database (Clinical Trials.gov, WHO International Clinical Trials Registry Platform, and Chinese Clinical Trials Registry) will also be scrutinized as to avoid missing studies, and ongoing or unpublished clinical trials.

The following search terms will be used in the search strategy: deep neck space abscesses, ultrasound, surgical, and randomized controlled trial. Related search terms will also be translated into Chinese for literature research and study identification in Chinese databases. The search strategies are listed in Supplementary Appendix file 1. To avoid missing published studies during the systematic review preparation, we will update the literature search results prior to the final publication of systematic reviews.

Data collection and analysis

Selection of studies

Two authors (NW and DL) will independently screen the potentially eligible studies by reading titles and abstracts. All identified and relevant

For peer review only - http://bmjopen.bmj.com/site/about/guidelines.xhtml

full-text publications will be retrieved from the database search process by screening the full text thoroughly, obviously ineligible records will be eliminated, and the reasons for exclusion will be recorded. Any disagreement on eligibility of studies will be resolved through discussion or arbitrated by a third review coauthor (Z-JQ). A fourth reviewer (Z-WY) will check out all procedures carefully prior to the final confirmation of the data extraction. Duplicate publications and companion papers of the same trial will be discussed by all review authors. The entire search process is displayed in the PRISMA flow diagram (figure 1).

Data extraction

Two review authors (NW and DL) will extract data from all included studies according to a standardized data collection form (Excel version 2013, Microsoft Inc, Washington DC, USA). The data extraction form included the publication information (the first author's name, author's country publication year), participants' demographic data (sample size; age; gender; BMI; ASA physical status classification levels; inclusion and exclusion criteria if necessary), locations of DNAs, experience of the operator, detailed information on drainage techniques (type of drainage techniques: surgical incision drainage or ultrasound-guided puncture drainage; days of drainage tube retention; and retreatment of multiple punctures or incisions), microbial cultures, antibiotic treatment(s) and any kind of outcomes including primary and secondary outcomes.

For peer review only

Study design characteristics, including the following domains: randomization, allocation concealment, blinding (patients, treatment providers, outcome investigators), incomplete outcome data collection and statistical analysis, and outcome reporting, will be recorded. Continuous data will be recorded as the mean± SD, and dichotomous data will be the recorded as percentages or proportions. A third review author (CG) will cross-check the data to ensure precision. We will contact the corresponding author of the research via email to acquire the missing or incomplete data for analysis as much as possible. In addition, the record will be excluded from the final review if no reply is received. Numerical data in the graphs will be extracted by Adobe Photoshop if necessary.[42] In addition, mean and standard deviation will be estimated from the median, mid-range, and/or mid-quartile range as described by Luo D and Wan X.[43,44]

Quality assessment

Risk of bias in each included study will be evaluated independently by two review authors (Z-YB and Z-WY) under the guidance of the Cochrane risk-of-bias tool 2 (RoB 2.0).[45] The methodology including random sequence generation, allocation concealment, blinding (blinding of participants, personnel, and outcome assessment), incomplete outcome data, selective outcome reporting, other risks of bias and overall risk of bias will be assessed. Each included study will be evaluated by the Cochrane risk of bias tool and then classified into three levels (low risk,

For peer review only - http://bmjopen.bmj.com/site/about/guidelines.xhtml

unclear risk and high risk of bias).[40,46] Any discrepancies will be settled through discussions by all review authors or arbitration by a third reviewer (CG). The assessment of risk of bias is listed in Supplementary Appendix fie 2.

Grading of Recommendations Assessment, Development, and Evaluation (GRADE) methodology will be used to assess the quality of the evidence for each outcome.[47] The quality of effect estimates will be classified as high, moderate, low or very low depending on the risk of bias, consistency, directness, precision, publication bias, large effect, and plausible confounding.[48] After the assessment, a table concerning the GRADE evidence profile will be created using GRADE profiler version 3.6.1 to rate the quality of all outcomes.

Measures of treatment effect

Mean differences (MDs) with 95% confidence intervals (CIs) and standardized mean differences (SMDs) with 95% confidence intervals (CIs) will be used for continuous outcome data with the same unit and different units, respectively. The relative risks (RRs) with 95% CIs will be used for dichotomous outcome data. Heterogeneity analyses will be performed by Review Manager version 5.4 (Rev Man, Cochrane Collaboration, Oxford, UK). Statistical heterogeneity will be assessed by the standard χ^2 test (α=0.1) and I^2 test. If $p \geq 0.1$ and if $I^2 \leq 50\%$, the fixed-effects model will be used. If $p < 0.1$ or $I^2 > 50\%$, the random-effects model will be applied.[40] A P

For peer review only - http://bmjopen.bmj.com/site/about/guidelines.xhtml

value <0.05 will be considered statistically significant.

Trial Sequential Analysis

The required information size (RIS) will be calculated to correct the risks of random errors by trial sequential analysis (TSA) using the TSA program version 0.9.5.10 Beta (Copenhagen Trial Unit, Copenhagen, Denmark).[48-50] TSA program version is available at http://www.ctu.dk/tsa.[51] Each outcome will be detected by the RIS, the cumulative Z-curve and the TSA monitoring boundaries.[52,53]

To calculate the RIS, the risk of type I error will be maintained at 5% with a power of 90% in both continuous and dichotomous outcomes.[54] For continuous outcomes, a mean difference of the observed SD/2 will be used to calculate the RIS. For dichotomous outcomes, the proportion or percentage from the control group, a relative risk reduction (RRR) of 20% or -20% will be used to calculate the RIS. When there is limited evidence available about the intervention under investigation, we will estimate a clinically relevant intervention effect by using clinical experience and evidence from related areas.[53-55]

For peer review only

Subgroup analysis

The results will be comprehensively interpreted through an analysis of subgroups or subsets as much as possible. If sufficient trials are available, data from different participants' ages, and different locations of DNAs will be analyzed independently.

For peer review only - http://bmjopen.bmj.com/site/about/guidelines.xhtml

► Different participants' ages (infants; children; adults; aged).

► Different locations of DNAs (parapharyngeal space, submandibular space, masseter space, retropharyngeal space, sublingual space, prevertebral space, and carotid space).

The interaction p value will be used to test the statistically significant subgroup difference. If a significant difference between subgroups exists (testing for interaction $p<0.05$), the results for individual subgroups will be reported separately.[40]

Assessment of publication biases

Visual judgment of the funnel plot asymmetry and Egger's regression test will be performed to estimate the potential publication bias, while more than 10 original studies involved the same outcome measure.[56,57] Analyses of reporting bias will be performed by Stata/MP 16.0 (Stata Corp, College Station, TX, USA). The effect sizes of each included study will be normally symmetrically distributed around the center of a funnel plot in the absence of publication bias.[56] In the case of publication bias, trim and fill analysis will be performed to estimate the number of missing studies that might exist and amend the bias.[58] A level of $P < 0.05$ will be considered statistically significant and indicated potential publication bias.

Grading the quality of evidence

The quality of evidence for each outcome will be assessed using the Grading of Recommendations Assessment, Development and Evaluation

For peer review only - http://bmjopen.bmj.com/site/about/guidelines.xhtml

(GRADE) criteria.[59] The quality of effect estimates will be classified as high, moderate, low or very low depending on the risk of bias, consistency, directness, precision and publication bias.[59] Data from randomized controlled trials will be classified as high-quality evidence according to GRADE. However, it can be degraded according to risk of bias, imprecision, inconsistency, indirectness or publication bias.

Patient and public involvement statement

Patients or the public were not involved in the design, conduct, reporting, or dissemination plans of our research.

DISCUSSION

Surgical incision-and-drainage with appropriate administration of antibiotics is the main treatment method for DNAs.[14-19] Due to the drawbacks and controversies of the surgical incision-and-drainage, the optimal drainage technique for the treatment of DNAs remains uncertain.[37-38] With the popularization of minimally invasive technology, image guided needle aspiration or drainage techniques have been testified to be an efficacious alternative treatment for some DNAs.[20-37] Randomized control trials with small sample sizes, suggested that image guided needle aspiration offers a reduced length of hospital stay and cost savings for some DNAs, compared to surgical incision and drainage.[25-29] In addition, the benefit of ultrasound-guided drainage for some DNAs was based on case reports, case series, reviews and retrospective studies.[20-24, 28-36] Due to the

For peer review only - http://bmjopen.bmj.com/site/about/guidelines.xhtml

limited current clinical evidence, the efficacy and safety of the ultrasound-guided drainage remains unclear.[37-38] This systematic review will provide an overview of the current state of evidence on the clinical efficacy and safety of ultrasound-guided drainage for DNAs. We will examine the length of hospital stay, cure rate, overall cost, and complications of ultrasound-guided drainage. The results of this systematic review will facilitate clinical decision making on better treatment of patients with DNAs.

This systematic review protocol was rigorously performed according to the Preferred Reporting Items for Systematic Review and Meta-Analyses Protocols (PRISMA-P) guidelines rigorously. The strengths of our systematic review are as follows: First, a comprehensive literature search of English databases, Chinese databases, and the trial registry database will be performed. Second, we will perform multivariable analysis (including subgroup analysis; trial sequential analysis for random errors; GRADE for study quality assessment; funnel plots and Egger's regression test for publication bias) to confirm the quality of the evidence. Third, numerical data in the graphs will be extracted and data that cannot be analyzed will be transformed.

Limitations are as follows: First, the experience of operators varied in the ultrasound-guided drainage and surgical incision drainage techniques, leading to potential heterogeneity. Second, the sample size of each

For peer review only - http://bmjopen.bmj.com/site/about/guidelines.xhtml

included study may be limited, and the number of studies with available data for subgroup analyses may be small. Third, studies with high-level evidence such as well-designed randomized controlled trials with double-blind designs may be limited, as it is difficult to perform blinding for different drainage techniques. Fourth, it is difficult to define a significant clinically plausible value of mean difference and relative risk increase/decrease during literature research or the clinical experience due to the limited current clinical evidence. Therefore, a significant clinically plausible value will be defined according to TSA guidelines.

ETHICS AND DISSEMINATION

Ethical approval was not required for this systematic review protocol. The findings will be disseminated through peer-reviewed publications.

Timelines

Formal screening of search results will begin in September 2023. Data extraction will begin in December 2023. The project will be completed in March 2024.

Author Contributions

Z-JQ and DL conceived the idea for this systematic review. All authors (NW, DL, CG, Z-YB, Z-WY, Z-JQ) developed the methodology for the systematic review. The manuscript was drafted by Z-JQ and CG, and revised by all authors. NW and DL will screen potential studies and extract the data. Z-YB and Z-WY will undertake risk of bias assessment

For peer review only

For peer review only - http://bmjopen.bmj.com/site/about/guidelines.xhtml

and assess the evidence quality. NW and Z-JQ will conduct the data synthesis. All authors contributed to the research and agreed to be responsible for all aspects of the work.

Funding

None.

Competing interests

None declared.

Data availability statement

Not applicable for this protocol.

Patient consent for publication

No patient was involved.

Provenance and peer review

Not commissioned; externally peer reviewed.

For peer review only

REFERENCES

1 Larawin V, Naipao J, Dubey SP. Head and neck space infections. *Otolaryngol Head Neck Surg* 2006; 135:889-93.

2 Santos Gorjón P, Blanco Pérez P, Morales Martín AC, *et al*. Deep neck infection. Review of 286 cases. *Acta Otorrinolaringol Esp* 2012; 63:31-41.

3 Almutairi DM, Alqahtani RM, Alshareef N, *et al*. Deep Neck Space Infections: A Retrospective Study of 183 Cases at a Tertiary Hospital. *Cureus* 2020;12: e6841.

4 Stalfors J, Adielsson A, Ebenfelt A, *et al*. Deep neck space infections remain a surgical challenge. A study of 72 patients. *Acta Otolaryngol* 2004; 124:1191-6.

5 Desa C, Tiwari M, Pednekar S, *et al*. Etiology and Complications of Deep Neck

For peer review only - http://bmjopen.bmj.com/site/about/guidelines.xhtml

Space Infections: A Hospital Based Retrospective Study. *Indian J Otolaryngol Head Neck Surg* 2023; 75:697-706.

6 Wang LF, Kuo WR, Tsai SM, *et al*. Characterizations of life threatening deep cervical space infections: A review of one hundred ninety-six cases. *Am J Otolaryngol* 2003; 24:111-7.

7 Nasir SB, Shuaibu IY, Labaran SA, *et al*. Management of Deep Neck Space Infections in a Tertiary Center in North West Nigeria. *Niger J Surg* 2019; 25:183-187.

8 Velhonoja J, Lääveri M, Soukka T, *et al*. Deep neck space infections: an upward trend and changing characteristics. *Eur Arch Otorhinolaryngol* 2020; 277:863-872.

9 Dalla Torre D, Brunold S, Kisielewsky I, *et al*. Life-threatening complications of deep neck space infections. *Wien Klin Wochenschr* 2013; 125:680-6.

10 Priyamvada S, Motwani G. A Study on Deep Neck Space Infections. *Indian J Otolaryngol Head Neck Surg* 2019; 71:912-917.

11 Lee YQ, Kanagalingam J. Deep neck abscesses: the Singapore experience. *Eur Arch Otorhinolaryngol* 2011; 268:609-14.

12 Potter JK, Herford AS, Ellis E 3rd. Tracheotomy versus endotracheal intubation for airway management in deep neck space infections. *J Oral Maxillofac Surg* 2002; 60:349-54;

13 Bhardwaj R, Makkar S, Gupta A, *et al*. Deep Neck Space Infections: Current Trends and Intricacies of Management? *Indian J Otolaryngol Head Neck Surg* 2022; 74:2344-2349.

For peer review only - http://bmjopen.bmj.com/site/about/guidelines.xhtml

14 Boscolo RP, Stellin M, Muzzi E, *et al*. Deep neck space infections: A study of 365 cases highlighting recommendations for management and treatment. *Eur Arch Otorhinolaryngol* 2012; 269: 1241-49.

15 Bakir S, Tanriverdi MH, Gun R, *et al*. Deep neck space infections: A retrospective review of 173 cases. *Am J Otolaryngol* 2012; 33:56-63.

16 Daramola OO, Flanagan CE, Maisel RH, *et al*. Diagnosis and treatment of deep neck space abscesses. *Otolaryngol Head Neck Surg* 2009; 141:123-30.

17 Sheikh Z, Yu B, Heywood E, *et al*. The assessment and management of deep neck space infections in adults: A systematic review and qualitative evidence synthesis. *Clin Otolaryngol* 2023; 48:540-562.

18 Wong DK, Brown C, Mills N, *et al*. To drain or not to drain-management of pediatric deep neck abscesses: a case-control study. *Int J Pediatr Otorhinolaryngol* 2012; 76:1810-3.

19 Carbone PN, Capra GG, Brigger MT. Antibiotic therapy for pediatric deep neck abscesses: a systematic review. *Int J Pediatr Otorhinolaryngol* 2012; 76:1647-53.

20 Joo BY, Jang AL, Lee JH, *et al*. Application of ultrasound-guided pigtail catheter drainage for abscesses in the head and neck. *Clin Otolaryngol* 2017; 42:1087-1091.

21 Baatenburg de Jong RJ, Rongen RJ, Lameris JS, *et al*. Ultrasound-guided percutaneous drainage of deep neck abscesses. *Clin Otolaryngol* 1990; 15:159-66.

22 Gaspari RJ, Sanseverino A, Gleeson T. Abscess Incision and Drainage With or Without Ultrasonography: A Randomized Controlled Trial. *Ann Emerg Med* 2019; 73:1-7.

For peer review only - http://bmjopen.bmj.com/site/about/guidelines.xhtml

23 Ochi K, Ogino S, Fukamizu K, *et al*. US-guided drainage of deep neck space abscess. *Acta Otolaryngol Suppl* 1996; 522:120-3.

24 Chang KP, Chen YL, Hao SP, *et al*. Ultrasound-guided closed drainage for abscesses of the head and neck. *Otolaryngol Head Neck Surg* 2005;132: 119-24.

25 Biron VL, Kurien G, Dziegielewski P, *et al*. Surgical vs ultrasound-guided drainage of deep neck space abscesses: a randomized controlled trial: surgical vs ultrasound drainage. *J Otolaryngol Head Neck Surg* 2013; 42:18.

26 Dabirmoghaddam P, Mohseni A, Navvabi Z, *et al*. Is ultrasonography-guided drainage a safe and effective alternative to incision and drainage for deep neck space abscesses? *J Laryngol Otol* 2017; 131:259-263.

27 Limardo A, Blanco L, Menéndez J, *et al*. Ultrasound-guided drainage vs surgical drainage of deep neck space abscesses: A randomized controlled trial. *Acta Otorrinolaringol Esp (Engl Ed)* 2022; 73:4-10.

28 Fan X, Tao S. Comparison of ultrasound-guided puncture drainage and incision drainage for deep neck abscess. *Gland Surg* 2021;10: 1431-1438.

29 Strassen U, Grimler C, Hofauer B. Ultrasound-Guided Needle Aspiration vs. Surgical Incision of Parotid Abscesses. *J Clin Med* 2022; 11:7425.

30 Delides A, Manoli E, Papadopoulos M, *et al*. Ultrasound-guided transoral drainage of a paediatric parapharyngeal abscess. *J Laryngol Otol* 2014; 128:1120-2.

31 Hagiwara Y, Araga T, Saito Y, *et al*. Utility of transoral pharyngeal ultrasonography for puncture drainage of peritonsillar abscess. *Clin Case Rep* 2019;7: 839-840.

For peer review only

For peer review only - http://bmjopen.bmj.com/site/about/guidelines.xhtml

32 Yeow KM, Liao CT, Hao SP. US-guided needle aspiration and catheter drainage as an alternative to open surgical drainage for uniloculated neck abscesses. *J Vasc Interv Radiol* 2001;12: 589-94.

33 Zeng DS, Chen JY, Yu D, *et al*. Application of Percutaneous Puncture in Maxillofacial and Cervical Space Infection with the Guide of B-mode Ultrasound. *Journal of Medical Research* 2019; 48:121-124.

34 Lin Y, An L, Li L. New treatment choice of head and neck cellulitis: multi-catheter drainage guided by ultrasound percutaneous puncture. *Chinese Journal of Otorhinolaryngology Head and Neck Surgery* 2017; 52:110-2.

35 Baek MY, Park KH, We JH, *et al*. Needle aspiration as therapeutic management for suppurative cervical lymphadenitis in children. *Korean J Pediatr* 2010; 53:801-4.

36 Serour F, Gorenstein A, Somekh E. Needle aspiration for suppurative cervical lymphadenitis. *Clin Pediatr (Phila)* 2002;41: 471-4.

37 Dunya G, Keefe KR, Orb Q, *et al*. Surgical vs interventional radiology drainage of neck abscesses in pediatric patients. *Int J Pediatr Otorhinolaryngol* 2023; 164:111382.

38 Lawrence R, Bateman N. Controversies in the management of deep neck space infection in children: an evidence-based review. *Clin Otolaryngol* 2017; 42:156-163.

39 Shamseer L, Moher D, Clarke M. Preferred reporting items for systematic review and meta-analysis protocols (PRISMA-P) 2015: elaboration and explanation. *BMJ*

For peer review only

2015; 350: g7647.

40 Higgins JPT, Thomas J, Chandler J, *et al*. Cochrane Handbook for Systematic Reviews of Interventions version 6.2 (updated February 2021). Cochrane, 2021. Available: *www.training.cochrane.org/handbook.*

41 Page MJ, McKenzie JE, Bossuyt PM, *et al*. The PRISMA 2020 statement: an updated guideline for reporting systematic reviews. *BMJ* 2021; 372: n71.

42 Gheibi S, Mahmoodzadeh A, Kashfi K, Jeddi S, Ghasemi A. Data Extraction from Graphs Using Adobe Photoshop: Applications for Meta-Analyses. *Int J Endocrinol Metab* 2019;17: e95216.

43 Luo D, Wan X, Liu J, Tong T. Optimally estimating the sample mean from the sample size, median, mid-range, and/or mid-quartile range. *Stat Methods Med Res 2018*; 27:1785-1805.

44 Wan X, Wang W, Liu J, Tong T. Estimating the sample mean and standard deviation from the sample size, median, range and/or interquartile range. *BMC Med Res Methodol* 2014; 14:135.

45 Sterne JAC, Savović J, Page MJ, *et al*. RoB 2: a revised tool for assessing risk of bias in randomised trials. *BMJ* 2019; 366: l4898.

46 Savović J, Turner RM, Mawdsley D, *et al*. Association between Risk of-Bias assessments and results of randomized trials in Cochrane reviews: the ROBES Meta-Epidemiologic study. *Am J Epidemiol* 2018;187: 1113-22.

47 Guyatt G, Oxman AD, Akl EA, *et al.* Grade guidelines: 1. Introduction-GRADE evidence profiles and summary of findings tables. *J Clin Epidemiol* 2011; 64:383-94.

For peer review only - http://bmjopen.bmj.com/site/about/guidelines.xhtml

48 Wetterslev J, Thorlund K, Brok J, *et al*. Estimating required information size by quantifying diversity in random-effects model meta-analyses. *BMC Med Res Methodol* 2009; 9:86.

49 Wetterslev J, Jakobsen JC, Gluud C. Trial sequential analysis in systematic reviews with meta-analysis. *BMC Med Res Methodol* 2017; 17:39.

50 Wetterslev J, Thorlund K, Brok J, *et al*. Trial sequential analysis may establish when firm evidence is reached in cumulative meta-analysis. *J Clin Epidemiol* 2008; 61: 64-75.

51 Thorlund K, Engstrøm J, Wetterslev J, *et al.* User manual for trial sequential analysis (TSA) Copenhagen trial unit, centre for clinical intervention research, Denmark; 2011.Available: http://www.ctu.dk/tsa [Accessed 2023].

52 Imberger G, Thorlund K, Gluud C, *et al*. False-Positive findings in Cochrane meta-analyses with and without application of trial sequential analysis: an empirical review. *BMJ Open* 2016;6: e011890.

53 Brok J, Thorlund K, Gluud C, *et al*. Trial sequential analysis reveals insufficient information size and potentially false positive results in many meta-analyses. *J Clin Epidemiol* 2008; 61:763-9.

54 Norman GR, Sloan JA, Wyrwich KW. Interpretation of changes in health-related quality of life: the remarkable universality of half a standard deviation. *Med Care* 2003; 41:582-92.

55 Miladinovic B, Mhaskar R, Hozo I, *et al*. Optimal information size in trial sequential analysis of time-to-event outcomes reveals potentially inconclusive

For peer review only - http://bmjopen.bmj.com/site/about/guidelines.xhtml

results because of the risk of random error. *J Clin Epidemiol* 2013; 66:654-9.

56 Egger M, Davey Smith G, Schneider M, *et al*. Bias in meta-analysis detected by a simple, graphical test. *BMJ* 1997;315: 629-34.

57 Sterne JA, Gavaghan D, Egger M. Publication and related bias in meta-analysis: power of statistical tests and prevalence in the literature. *J Clin Epidemiol* 2000; 53:1119-29.

58 Mavridis D, Salanti G. How to assess publication bias: funnel plot, trim-and-fill method and selection models. *Evid Based Ment Health* 2014; 17:30.

59 Guyatt G, Oxman AD, Akl EA, *et al.* Grade guidelines: 1. Introduction-GRADE evidence profiles and summary of findings tables. *J Clin Epidemiol* 2011; 64:383-94.

Figure Legends

Figure 1. The PRISMA flow diagram. PRISMA, Preferred Reporting Items for Systematic Reviews and Meta-analysis. *Consider, if feasible to do so, reporting the number of records identified from each database or register searched (rather than the total number across all databases/registers). **If automation tools were used, indicate how many records were excluded by a human and how many were excluded by automation tools.

For peer review only

For peer review only - http://bmjopen.bmj.com/site/about/guidelines.xhtml

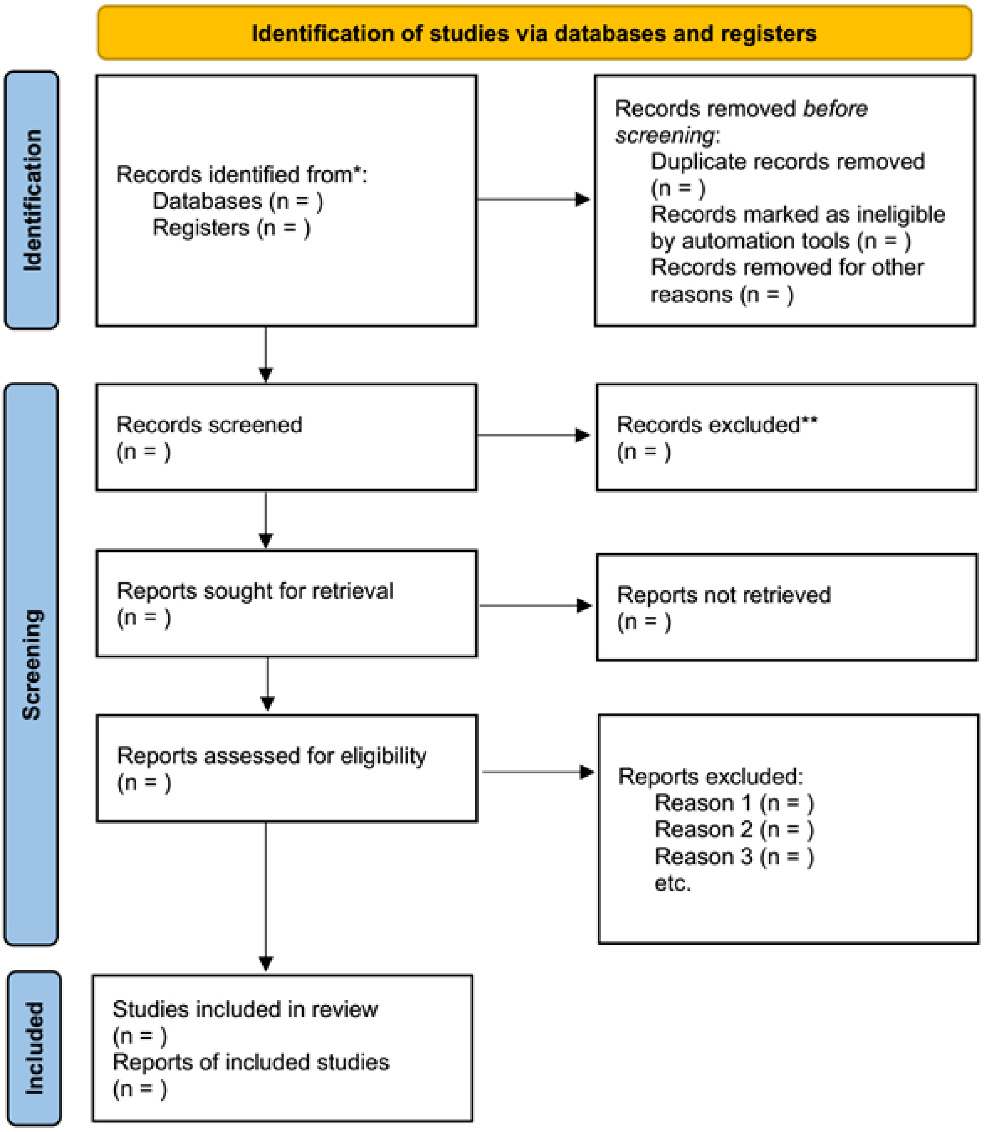

The PRISMA flow diagram

157x180mm (96 x 96 DPI)

Supplementary Appendix file 1: Search strategy

Search strategy of PubMed as follows:

#1 "deep"[All Fields] AND ("neck"[MeSH Terms] OR "neck"[All Fields]) AND ("space"[All Fields] OR "space s"[All Fields] OR "spaces"[All Fields])) OR "deep neck space"[Title/Abstract] "parapharyngeal space"[Title/Abstract] OR "submandibular space"[Title/Abstract] OR "masseter space"[Title/Abstract] OR "retropharyngeal space "[Title/Abstract] OR "sublingual space "[Title/Abstract] OR "prevertebral space "[Title/Abstract] OR "carotid space"[Title/Abstract]

#2 "infections"[MeSH Terms] OR "infection" [Text Word] OR "infections" [Text Word] OR "infections"[Title/Abstract] OR "infection"[Title/Abstract]

#3 "abscess"[MeSH Terms] OR "abscess " [Text Word] OR "abscesses" [Text Word] OR "abscess " [Title/Abstract] OR "abscesses "[Title/Abstract]

#4 #2 OR #3

#5 #1 and #4

#6 "Ultrasonography, Interventional"[Mesh] OR "Ultrasonics"[Mesh] OR "Ultrasonography"[Mesh] OR "ultrasound-guided "[Title/Abstract]

#7 "Surgical Procedures, Operative"[Mesh] OR ("Surgical"[All Fields] AND "procedures"[All Fields] AND "operative"[All Fields]) OR "operative surgical procedures"[All Fields] OR "Surgical"[All Fields] OR "surgically"[All Fields] OR "surgicals"[All Fields]) AND "Surgical"[Title/Abstract]

#8 "Randomized Controlled Trial" [Publication Type] OR "Randomized Controlled Trials as Topic"[Mesh] OR "Randomized Controlled Trial, Veterinary" [Publication Type] OR "Controlled Clinical Trial" [Publication Type] OR “randomized” [Title/Abstract] OR “randomized” [Title/Abstract] OR “Placebo” [Title/Abstract] OR “randomly” [Title/Abstract] OR “Clinical trial” [Title]

#9 #5 and #6 and #7 and #8

Search strategy of Cochrane library as follows:

#1 (deep neck space or parapharyngeal space or submandibular space or masseter space or retropharyngeal space or sublingual space or prevertebral space or carotid space): ti,ab,kw

#2 MeSH descriptor: Abscess Explode all trees

#3 (abscess): ti,ab,kw

#4 #2or #3

#5 MeSH descriptor: Infections Explode all trees

#6 (infections): ti,ab,kw

#7 #5or #6

#8 #4or #7

#9 #1 and #8

#10 MeSH descriptor: Ultrasonography Explode all trees

#11 (ultrasound-guided): ti,ab,kw

#12 #10 or #11

#13MeSH descriptor: Surgical Procedures, Operative Explode all trees

#14 (surgical): ti,ab,kw

#15 #13or #14

#16 (controlled clinical trial):pt or (randomized controlled trial):pt or (random*): ti,ab,kw or (Clinical trial):ti,ab,kw

#17 #9 and #12 and #15 and #16

Search strategy of Web of Science as follows:

#1 TS= (deep neck space or parapharyngeal space or submandibular space or masseter space or retropharyngeal space or sublingual space or prevertebral space or carotid space)

#2 TS= (infection)

#3 TS= (abscess)

#4 #2 or #3

#5 #1and #4

#6 TS= (surgical)

#7 TS= (ultrasound)

#8 #6 and #7

#9 TS= (random* or Clinical trial)

#10 #5 and #8 and #9

Search strategy for Ovid Medline as follows:

#1(deep neck space or parapharyngeal space or submandibular space or masseter space or retropharyngeal space or sublingual space or prevertebral space or carotid space).mp.

For peer review only

#2 exp infections/

#3 (infections).mp.

#4 #2 or # 3

#5 exp abscess /

#6 (abscess).mp.

#7 #5 or #6

#8 #4 or #7

#9 #1 and #8

#10 exp Surgical/

#11 (Surgical).mp.

#12 #10 or #11

#13 exp ultrasound/

#14 (ultrasound).mp.

#15 #13 or #14

#16 #12 and #15

#17 randomized controlled trial.pt.

#18 controlled clinical trial.pt.

#19 randomized.ab.

#20 placebo.ab.

#21 clinical trials as topic.sh.

#22 randomly.ab.

#23 trial.ti.

#24 #17 or #18 or #19 or #20 or #21 or #22 or #23

#25 #9 and #16 and #24

Search strategy for Embase as follows:

#1 (deep neck space or parapharyngeal space or submandibular space or masseter space or retropharyngeal space or sublingual space or prevertebral space or carotid space) .mp.

#2 exp Abscess/

#3 (abscess).mp.

#4 #2 or # 3

For peer review only

#5 exp infections /

#6 (infections).mp.

#7 #5 or # 6

#8 #4 or # 7

#9 #1 and #8

#10 exp ultrasonography /

#11 (ultrasound-guided).mp.

#12 #10 or # 11

#13 #9 and #12

#14 exp Surgical Procedures /

#15 (Surgical).mp.

#16 #14 or # 15

#17 exp randomized controlled trial/

#18 (random*).mp.

#19 (placebo*).mp.

#20 Clinical trial.mp.

#21 clinical trials as topic.sh.

#22 #17 or #18 or #19 or #20 or #21

#23 #13 and #16 and #22

WHO ICTRP Trial registry

http://apps.who.int/trialsearch (WHO ICTRP register) will be searched via the advanced search page. Search terms were: (deep neck space OR parapharyngeal space OR submandibular space OR masseter space OR retropharyngeal space OR sublingual space OR prevertebral space OR carotid space) AND (abscess OR infections) AND (ultrasound).

Clinicaltrials.gov search strategy

http://clinicaltrials.gov (NIH register) will be searched via advanced search page. Search terms were: Condition or disease: (deep neck space OR parapharyngeal space OR submandibular space OR masseter space OR retropharyngeal space OR sublingual space OR prevertebral space OR carotid space) AND (abscess OR infections);

Study type: Interventional Study (clinical trial);

For peer review only

Intervention/treatment: (ultrasound-guided);

Chinese Clinical Trial Registry

https://www.chictr.org.cn/index.html (Chinese Clinical Trial Registry, ChiCTR) will be searched via Subject of Registration. Search terms were: 颈部脓肿；下颌脓肿；扁桃体周围脓肿；咽旁间隙脓肿；颌下间隙脓肿；咬肌间隙脓肿；咽后间隙脓肿；舌下间隙脓肿；椎前间隙脓肿；颈动脉间隙脓肿；颈部感染；下颌感染；扁桃体周围感染；咽旁间隙感染；颌下间隙感染；咬肌间隙感染；咽后间隙感染；舌下间隙感染；椎前间隙感染；颈动脉间隙感染；

Chinese database

China National Knowledge Infrastructure (CNKI) search strategy

(颈部[全部字段]or 下颌[全部字段]or 扁桃[全部字段]or 咽旁[全部字段]or 颌下[全部字段]or 咬肌[全部字段]or 咽后[全部字段]or 舌下[全部字段]or 椎前[全部字段]or 颈动脉 [全部字段]) and (脓肿[全部字段] or 感染[全部字段]) and (超声[全部字段] or B 超[全部字段]) and (随机[全部字段] or 对照[全部字段])

Chinese BioMedical Literature (CBM)

(颈部[全部字段]or 下颌[全部字段]or 扁桃[全部字段]or 咽旁[全部字段]or 颌下[全部字段]or 咬肌[全部字段]or 咽后[全部字段]or 舌下[全部字段]or 椎前[全部字段]or 颈动脉 [全部字段]) and (脓肿[全部字段] or 感染[全部字段]) and (超声[全部字段] or B 超[全部字段]) and (随机[全部字段] or 对照[全部字段])

VIP database

关键词=(颈部 or 下颌 or 扁桃 or 咽旁 or 颌下 or 咬肌 or 咽后 or 舌下 or 椎前 or 颈动脉) AND 关键词= (脓肿 or 感染) AND 关键词= (超声 or B 超) AND 关键词= (随机 or 对照)

Wan fang database.

(颈部[全部字段]or 下颌[全部字段]or 扁桃[全部字段]or 咽旁[全部字段]or 颌下[全部字段]or 咬肌[全部字段]or 咽后[全部字段]or 舌下[全部字段]or 椎前[全部字段]or 颈动脉 [全部字段])

For peer review only

and (脓肿[全部字段] or 感染[全部字段]) and (超声[全部字段] or B 超[全部字段]) and (随机[全部字段] or 对照[全部字段])

For peer review only

Supplementary Appendix file 2 : Assessment of risk of bias

Random sequence generation

- ***Low risk:*** If sequence generation was achieved using computer random number generator or a random number table. Drawing lots, tossing a coin, shuffling cards, and throwing dice were also considered adequate if performed by an independent adjudicator.
- ***Unclear risk:*** If the method of randomisation was not specified, but the trial was still presented as being randomised.
- ***High risk:*** If the allocation sequence is not randomised or only quasi-randomised. These trials will be excluded.

Allocation concealment

- ***Low risk:*** If the allocation of patients was performed by a central independent unit, onsite locked computer or identical-looking numbered sealed envelopes.
- ***Uncertain risk:*** If the trial was classified as randomised but the allocation concealment process was not described.
- ***High risk:*** If the allocation sequence was familiar to the investigators who assigned participants.

Blinding of participants and treatment providers

- ***Low risk:*** If the participants and the treatment providers were blinded to intervention allocation and this was described.
- ***Uncertain risk:*** If the procedure of blinding was insufficiently described.
- ***High risk:*** If blinding of participants and the treatment providers was not performed.

Blinding of outcome assessment

- ***Low risk of bias:*** If it was mentioned that outcome assessors were blinded and this was described.
- ***Uncertain risk of bias:*** If it was not mentioned if the outcome assessors in the trial were blinded or the extent of blinding was insufficiently described.
- ***High risk of bias:*** If no blinding or incomplete blinding of outcome assessors was performed.

Incomplete outcome data

- ***Low risk of bias:*** If missing data were unlikely to make treatment effects depart from plausible

For peer review only

values. This could be either (1) there were no drop-outs or withdrawals for all outcomes, or (2) the numbers and reasons for the withdrawals and drop-outs for all outcomes were clearly stated and could be described as being similar to both groups. Generally, the trial is judged as at a low risk of bias due to incomplete outcome data if drop-outs are less than 5%. However, the 5% cut-off is not definitive.

- ***Uncertain risk of bias:*** If there was insufficient information to assess whether missing data were likely to induce bias on the results.
- ***High risk of bias:*** If the results were likely to be biased due to missing data either because the pattern of drop-outs could be described as being different in the two intervention groups or the trial used improper methods in dealing with the missing data (e.g. last observation carried forward).

Selective outcome reporting

- ***Low risk of bias:*** If a protocol was published before or at the time the trial was begun and the outcomes specified in the protocol were reported on. If there is no protocol or the protocol was published after the trial has begun, reporting of serious adverse events will grant the trial a grade of low risk of bias.
- ***Uncertain risk of bias:*** If no protocol was published and the outcome of serious adverse events were not reported on.
- ***High risk of bias:*** If the outcomes in the protocol were not reported on.

Other risks of bias

- ***Low risk of bias:*** If the trial appears to be free of other components that could put it at risk of bias.
- ***Unclear risk of bias:*** If the trial may or may not be free of other components that could put it at risk of bias.
- ***High risk of bias:*** If there are other factors in the trial that could put it at risk of bias (including, Design-specific risk of bias, stopped early due to some data-dependent process including a formal-stopping rule, baseline imbalance, claimed fraudulent, blocked randomization in unblinded trials, differential diagnostic activity, contamination, inappropriate measurement instrument for outcomes, deviation from the study protocol unrelated to the clinical practice, authors conducted trials on the same topic, academic bias, for-profit bias, inappropriate

For peer review only

financial conflict of interest).

Overall risk of bias

- ***Low risk of bias:*** The trial will be classified as overall 'low risk of bias' only if all of the bias domains described in the above paragraphs are classified as 'low risk of bias'.
- ***High risk of bias:*** The trial will be classified as 'high risk of bias' if any of the bias risk domains described in the above are classified as 'unclear' or 'high risk of bias'.
- We will assess the domains 'blinding of outcome assessment', 'incomplete outcome data', and 'selective out- come reporting' for each outcome result. Thus, we can assess the bias risk for each outcome assessed in addition to each trial. Our primary conclusions will be based on the results of our primary outcome results with overall low risk of bias. Both our primary and secondary conclusions will be presented in the summary of findings tables.

Criteria classification

- If all risk of bias domains were scored as having a low risk of bias, the trial was defined as having a low overall risk of bias.
- If one or more of the bias domains were scored as unclear or high risk of bias, the trial was defined as having a high overall risk of bias.
- Trials with a low risk of bias in all domains (including sequence generation, allocation concealment, blinding, incomplete data, selective outcome reporting, and other risks of bias) will be classified as having a low overall risk of bias.
- Trials with one or more of these domains scored as unclear or high risk of bias will be defined as having a high overall risk of bias.

For peer review only

PRISMA-P checklist

Table PRISMA-P (preferred reporting items for systematic review and meta-analysis protocols) 2015 checklist: recommended items to address in a systematic review protocol

Section and topic	Item No	Checklist item	Reported on page #
**Administrative information**			
Title:			
Identification	1a	Identify the report as a protocol of a systematic review	1
Update	1b	If the protocol is for an update of a previous systematic review, identify as such	None
Registration	2	If registered, provide the name of the registry (such as PROSPERO) and registration number	3,7
Authors:			
Contact	3a	Provide name, institutional affiliation, e-mail address of all protocol authors; provide physical mailing address of corresponding author	1
Contributions	3b	Describe contributions of protocol authors and identify the guarantor of the review	17-18
Amendments	4	If the protocol represents an amendment of a previously completed or published protocol, identify as such and list changes; otherwise, state plan for documenting important protocol amendments	None
Support:			
Sponsor	5b	Provide name for the review funder and/or sponsor	None
Role of sponsor or funder	5c	Describe roles of funder(s), sponsor(s), and/or institution(s), if any, in developing the protocol	None
**Introduction**			
Rationale	6	Describe the rationale for the review in the context of what is already known	4-6
Objectives	7	Provide an explicit statement of the question(s) the review will address with reference to participants, interventions, comparators, and outcomes (PICO)	6

**Methods**			
Eligibility criteria	8	Specify the study characteristics (such as PICO, study design, setting, time frame) and report characteristics (such as years considered, language, publication status) to be used as criteria for eligibility for the review	7-9
Information sources	9	Describe all intended information sources (such as electronic databases, contact with study authors, trial registers or other grey literature sources) with planned dates of coverage	8-11
Search strategy	10	Present draft of search strategy to be used for at least one electronic database, including planned limits, such that it could be repeated	8-9, S1
Study records:			
Data management	11a	Describe the mechanism(s) that will be used to manage records and data throughout the review	9-11
Selection process	11b	State the process that will be used for selecting studies (such as two independent reviewers) through each phase of the review (that is, screening, eligibility and inclusion in meta-analysis)	14-15
Data collection process	11c	Describe planned method of extracting data from reports (such as piloting forms, done independently, in duplicate), any processes for obtaining and confirming data from investigators	10-11
Data items	12	List and define all variables for which data will be sought (such as PICO items, funding sources), any pre-planned data assumptions and simplifications	10-11
Outcomes and prioritization	13	List and define all outcomes for which data will be sought, including prioritization of main and additional outcomes, with rationale	8
Risk of bias in individual studies	14	Describe anticipated methods for assessing risk of bias of individual studies, including whether this will be done at the outcome or study level, or both; state how this information will be used in data synthesis	11-15
Data synthesis	15a	Describe criteria under which study data will be quantitatively synthesised	12-14
	15b	If data are appropriate for quantitative synthesis, describe planned summary measures, methods of handling data and methods of combining data from studies, including any planned exploration of consistency (such as I^2, Kendall's τ)	12-14
	15c	Describe any proposed additional analyses (such as sensitivity or subgroup analyses, meta-regression)	13-14
	15d	If quantitative synthesis is not appropriate, describe the type of summary planned	12-13
Meta-bias(es)	16	Specify any planned assessment of meta-bias(es) (such as publication bias across studies, selective reporting within studies)	12-13

Confidence in cumulative evidence	17	Describe how the strength of the body of evidence will be assessed (such as GRADE)	14-15

For peer review only

BMJ Open

Ultrasound-guided puncture drainage vs surgical incision drainage for deep neck space abscesses: a protocol for a systematic review with meta-analysis and trial sequential analysis

Journal:	*BMJ Open*
Manuscript ID	bmjopen-2023-077631.R1
Article Type:	Protocol
Date Submitted by the Author:	29-Nov-2023
Complete List of Authors:	Nie, Wei; Sichuan University West China Hospital, Department of Anesthesiology<br>Du, Li; Sichuan Cancer Hospital and Research Institute, Department of Anesthesiology<br>Chen, Guo; Sichuan University West China Hospital, Department of Anesthesiology<br>Zheng, Yongbo; Sichuan University West China Hospital, Department of Otorhinolaryngology, Head & Neck Surgery<br>Yang, Xibiao; West China Hospital of Sichuan University, Department of Radiology<br>Li, Bo; West China Hospital of Sichuan University, Department of Otorhinolaryngology, Head & Neck Surgery<br>Zhang, Weiyi; Sichuan University West China Hospital, Department of Anesthesiology<br>Zheng, Jianqiao; Sichuan University West China Hospital, Department of Anesthesiology
**Primary Subject Heading**:	Ear, nose and throat/otolaryngology
Secondary Subject Heading:	Ear, nose and throat/otolaryngology, Evidence based practice, Medical management
Keywords:	Ultrasound < RADIOLOGY & IMAGING, Randomized Controlled Trial, Head & neck surgery < OTOLARYNGOLOGY

SCHOLARONE™
Manuscripts

TITLE PAGE

Ultrasound-guided puncture drainage vs surgical incision drainage for deep neck space abscesses: a protocol for a systematic review with meta-analysis and trial sequential analysis

Authors

Wei Nie[1] E-mail: nieweione@163.com
Li Du[2] E-mail: huaying-duli@163.com
Guo Chen[1] E-mail: Anesthesiology_SCU@163.com
Yongbo Zheng[3] E-mail: zheng_yongbo@126.com
Xibiao Yang[4] E-mail: yang061528@163.com
Bo Li[3] E-mail: liboent@wchscu.cn
Weiyi Zhang[1] E-mail: zhangweiyi@wchscu.cn
*Jianqiao Zheng[1] E-mail: zhjq1983@163.com

[1] Department of Anesthesiology, West China Hospital, Sichuan University No. 37th, Guoxue Alley, Wuhou District, Chengdu, Sichuan, China.

[2] Department of Anesthesiology, Sichuan Cancer Hospital & Institute, Sichuan Cancer Center, School of Medicine, University of Electronic Science and Technology of China, No.55th, People's South Road, Chengdu, Sichuan, China.

[3] Department of Otorhinolaryngology, Head & Neck Surgery, West China Hospital, Sichuan University, No. 37th, Guoxue Alley, Wuhou District, Chengdu, Sichuan, China.

[4] Department of Radiology, West China Hospital, Sichuan University, No. 37th, Guoxue Alley, Wuhou District, Chengdu, Sichuan, China.

*Corresponding Author
Name: Jianqiao Zheng
E-mail: zhjq1983@163.com
Phone: +86-28-8542-3593
Address: Department of Anesthesiology, Sichuan University West China Hospital, No. 37th, Guoxue Alley, Wuhou District, Chengdu 610041, Sichuan, China.

Words: 3010

For peer review only

ABSTRACT

Introduction Deep neck space abscesses (DNAs) are serious surgical emergencies, associated with life-threatening complications. Surgical incision-and-drainage combined with antibiotics is the main treatment for DNAs, but drawbacks still exist. Ultrasound-guided puncture drainage is an alternative treatment for some DNAs with limited clinical evidence. Hence, the optimal drainage technique for the treatment of DNAs remains unclear. Therefore, we will perform a protocol for a systematic review and meta-analysis to identify the efficacy of ultrasound-guided puncture drainage for DNAs.

Methods and analysis PubMed, Ovid Medline, Cochrane Library, Embase, Web of Science, China National Knowledge Infrastructure, Wanfang database, VIP database and trial registry databases will be searched from inception to September 2023 to identify randomized controlled trials of patients diagnosed DNAs accepting ultrasound-guided puncture drainage. Primary outcome will be the length of hospital stay. Secondary outcomes will be the cure rate, incidence of retreatment, complications and overall cost to the healthcare system. Fixed-effects or random-effects model will be used according to the statistical heterogeneity. Mean differences or standardized mean differences with 95% confidence intervals (CIs) for continuous data and risk ratio (RR) with 95% Cis for dichotomous data. The Cochrane risk-of-bias tool 2, Grading of

For peer review only - http://bmjopen.bmj.com/site/about/guidelines.xhtml

For peer review only

Recommendations Assessment, Development and Evaluation (GRADE) and trial sequential analysis will be conducted to evaluate the evidence quality and control the random errors. Funnel plots and Egger's regression test will be performed to evaluate publication bias.

Ethics and dissemination Ethical approval was not required for this systematic review protocol. The results will be disseminated through peer-reviewed publications.

Keywords deep neck space, abscesses, infection, ultrasound, surgical, meta-analysis, randomized controlled trial.

PROSPERO registration number CRD42023441031

Strengths and limitations of the study

► This research will be the first meta-analysis to compare the efficacy and safety of ultrasound-guided puncture drainage vs surgical incision drainage for deep neck space abscesses.

► Trial sequential analysis to control random errors of all outcomes.

► Data in the graphs will be extracted and data that cannot be analyzed will be transformed.

► Subgroup analysis based on participants' ages, and different locations of DNAs for heterogeneity assessment.

► The study will be limited to small sample size of each included study.

For peer review only - http://bmjopen.bmj.com/site/about/guidelines.xhtml

INTRODUCTION

Deep neck space abscesses (DNAs) are serious otolaryngology-head and neck surgery emergencies, defined as bacterial infections in the potential spaces and fascial planes of the neck, which could result in significant morbidity with potential mortality. [1-4] Dental infection is the most common cause of DNAs, and the prevalence of DNAs has been reduced with the advent of broad-spectrum antibiotics. [5] However, DNAs remains serious, spreads rapidly, and is associated with life-threatening complications.[6-9]Common and potentially life-threatening complications range from 15% to 35% in patients with DNAs, [5 10 11] including airway compromise, descending mediastinitis, pericarditis, pneumonia, pleural empyema, carotid arterial erosion, meningitis, sepsis, respiratory distress, jugular vein thrombosis, disseminated intravascular coagulation, and extracranial or intracranial extension of infection. [5 8-13] Therefore, the early detection of abscesses and thorough treatment are crucial.

Traditionally, the main treatment method for DNAs is complete surgical incision-and-drainage with appropriate administration of antibiotics. [14-19] However, surgical drainage has several drawbacks. First, the risk of inadvertent injuries to neighboring vital structures (including the internal jugular vein, carotid artery and cranial nerves) is associated with devastating complications. Second, surgical trauma danger

For peer review only - http://bmjopen.bmj.com/site/about/guidelines.xhtml

is increased as the inflammatory anatomy tissue is obscure and easily bled during incision when the abscess is deep. Third, patients suffering from surgical trauma stress become frail and more sensitive to pathogenic bacteria in the environment, increasing the risk of infection-related complications. Fourth, tumor-related DNAs could cause tumor dissemination during open surgical incision-and-drainage. Fifth, surgical incision-and-drainage was performed under general anesthesia in most circumstances, which will increase the anesthesia-related risk, especially in poor medical condition DNAs patients. Finally, surgical incision scars increase the risk of incision disunion and cause aesthetic defects. Therefore, a noninvasive, effective alternative to open surgical incision drainage may be beneficial to DNAs patients.

Minimally invasive techniques, such as ultrasound-guided puncture drainage, have been used to treat abscesses of the head and neck, even some DNAs. [20-37] Ultrasound-guided puncture drainage can avoid the above drawbacks of surgical incision drainage, and provide several advantages. First, ultrasound-guided puncture drainage can accurately locate the DNAs, eliminating trauma to the neighboring vital structures, and avoiding tumor dissemination during surgical incision. Second, the drainage tube can be placed under the real-time guidance of ultrasound, which is beneficial for thoroughly draining the abscess. Third, the real-time capability of ultrasound could facilitate dynamic observation after drainage and

For peer review only - http://bmjopen.bmj.com/site/about/guidelines.xhtml

monitoring the adequacy of drainage. Finally, ultrasound-guided puncture drainage does not cause skin nonunion or aesthetic defects. Several studies have shown that ultrasound-guided puncture drainage is a more effective option than surgical incision drainage for some DNAs. [20-36] In addition, ultrasound-guided puncture drainage can reduce the length of hospital stay. [25-29] However, most of these studies were case series, reviews and retrospective studies, and prospective and randomized controlled trials are rare.[20-36] In this situation, controversies still exist in the selection of drainage technique for the treatment of DNAs. [37 38]

Therefore, it is necessary to conduct a systematic review and meta-analysis to analyze the clinical efficacy of ultrasound-guided puncture drainage for DNAs. The outcomes of this systematic review will provide evidence for better clinical decision making and possible future directions for further clinical trials.

Objectives

We are performing this systematic review protocol with meta-analysis and trial sequential analysis (TSA) of randomized clinical trials to evaluate the clinical efficacy and safety of ultrasound-guided puncture drainage for DNAs.

METHODS AND ANALYSIS

Design and registration of the review

We devised this protocol according to the Preferred Reporting Items

For peer review only - http://bmjopen.bmj.com/site/about/guidelines.xhtml

for Systematic Reviews and Meta-Analyses Protocols (PRISMA-P) guidelines that have been registered with PROSPERO 2023 (registration number: CRD42023441031). [39] We will perform this systematic review and meta-analysis based on the Cochrane Handbook and report the results following the PRISMA statement. [40 41] This study is anticipated to begin searching in September 2023 and will be completed in March 2024.

Patient and public involvement statement

Patients or the public were not involved in the design, conduct, reporting, or dissemination plans of our research.

Inclusion criteria for study selection

Types of studies

Only randomized controlled trials (RCTs) involving the clinical efficacy of ultrasound-guided puncture drainage for DNAs will be included. There will be no language restrictions.

The exclusion criteria were as follows: (1) studies comparing ultrasound-guided needle puncture aspiration and ultrasound-guided catheter drainage or studies comparing ultrasound-guided needle puncture aspiration with other minimally invasive techniques (CT-guided needle puncture aspiration); (2) studies with data that could not be used for statistical analysis after data transformation, or studies with incomplete data, or original data that could not be obtained after contacting the corresponding authors; and (3) duplicate publications, letters or editorials,

For peer review only - http://bmjopen.bmj.com/site/about/guidelines.xhtml

abstracts from conferences, and reviews.

Types of participants

Participants diagnosed DNAs who accept ultrasound-guided puncture drainage will be included. There will be no limitations on participants' age, gender, ethnicity, body mass index (BMI) or American Society of Anesthesiologists (ASA) classification.

Types of interventions/controls

The intervention group will be the participants with DNAs accepting any kind of ultrasound-guided puncture drainage, while the control group will receive any kind of surgical incision drainage for DNAs.

Types of outcome measures

Primary outcomes

The primary outcome will be the length of hospital stay.

Secondary outcomes

1. The cure rate;
2. Incidence of retreatment (including secondary puncture drainage or incision drainage);
3. Incidence of complications: any kind of complications will be included. Complication criteria included drainage-related mechanical injury (vascular injury, nerve injury, airway injury, and bleeding), infectious complications (sepsis, mediastinal infection, pericarditis, pneumonia, arterial infection), airway obstruction, jugular vein thrombosis, skin

For peer review only - http://bmjopen.bmj.com/site/about/guidelines.xhtml

nonunion and other puncture complications;

4. Overall cost to the healthcare system;

Search strategy

Two reviewers (NW and DL) will independently perform the search and any disagreements will be arbitrated by a third reviewer (Z-WY). English and Chinese electronic databases will be searched from inception to September 2023 for published literature. English databases (including PubMed, Ovid Medline, Cochrane Library, Embase and Web of Science) and Chinese databases [China National Knowledge Infrastructure (CNKI), Wanfang database and VIP Database] will be searched. In addition, reference lists of each study and the trial registry database (Clinical Trials.gov, WHO International Clinical Trials Registry Platform, and Chinese Clinical Trials Registry) will also be scrutinized as to avoid missing studies, and ongoing or unpublished clinical trials.

The following search terms will be used in the search strategy: deep neck space abscesses, ultrasound, surgical, and randomized controlled trial. Related search terms will also be translated into Chinese for literature research and study identification in Chinese databases. The search strategies are listed in Supplementary Appendix file 1. To avoid missing published studies during the systematic review preparation, we will update the literature search results prior to the final publication of systematic reviews.

For peer review only - http://bmjopen.bmj.com/site/about/guidelines.xhtml

Data collection and analysis

Selection of studies

Two authors (NW and DL) will independently screen the potentially eligible studies by reading titles and abstracts. All identified and relevant full-text publications will be retrieved from the database search process by screening the full text thoroughly, obviously ineligible records will be eliminated, and the reasons for exclusion will be recorded. Any disagreement on eligibility of studies will be resolved through discussion or arbitrated by a third review coauthor (Z-JQ). A fourth reviewer (Z-WY) will check out all procedures carefully prior to the final confirmation of the data extraction. Duplicate publications and companion papers of the same trial will be discussed by all review authors. The entire search process is displayed in the PRISMA flow diagram (figure 1).

Data extraction

Two review authors (NW and DL) will extract data from all included studies according to a standardized data collection form (Excel version 2013, Microsoft Inc, Washington DC, USA). The data extraction form included the publication information (the first author's name, author's country publication year), participants' demographic data (sample size; age; gender; BMI; ASA physical status classification levels; inclusion and exclusion criteria if necessary), locations of DNAs, experience of the operator, detailed information on drainage techniques (type of drainage

For peer review only - http://bmjopen.bmj.com/site/about/guidelines.xhtml

techniques: surgical incision drainage or ultrasound-guided puncture drainage; days of drainage tube retention; and retreatment of multiple punctures or incisions), microbial cultures, antibiotic treatment(s) and any kind of outcomes including primary and secondary outcomes.

Study design characteristics, including the following domains: randomization, allocation concealment, blinding (patients, treatment providers, outcome investigators), incomplete outcome data collection and statistical analysis, and outcome reporting, will be recorded. Continuous data will be recorded as the mean± SD, and dichotomous data will be the recorded as percentages or proportions. A third review author (CG) will cross-check the data to ensure precision. We will contact the corresponding author of the research via email to acquire the missing or incomplete data for analysis as much as possible. In addition, the record will be excluded from the final review if no reply is received. Numerical data in the graphs will be extracted by Adobe Photoshop if necessary. [42] In addition, mean and standard deviation will be estimated from the median, mid-range, and/or mid-quartile range as described by Luo D and Wan X. [43 44]

Quality assessment

Risk of bias in each included study will be evaluated independently by two review authors (Z-YB and Z-WY) under the guidance of the Cochrane risk-of-bias tool 2 (RoB 2.0). [45] The methodology including random sequence generation, allocation concealment, blinding (blinding of

For peer review only - http://bmjopen.bmj.com/site/about/guidelines.xhtml

participants, personnel, and outcome assessment), incomplete outcome data, selective outcome reporting, other risks of bias and overall risk of bias will be assessed. Each included study will be evaluated by the Cochrane risk of bias tool and then classified into three levels (low risk, unclear risk and high risk of bias). [40 46] Any discrepancies will be settled through discussions by all review authors or arbitration by a third reviewer (CG). The assessment of risk of bias is listed in Supplementary Appendix fie 2.

Grading of Recommendations Assessment, Development, and Evaluation (GRADE) methodology will be used to assess the quality of the evidence for each outcome. [47] The quality of effect estimates will be classified as high, moderate, low or very low depending on the risk of bias, consistency, directness, precision, publication bias, large effect, and plausible confounding. [48] After the assessment, a table concerning the GRADE evidence profile will be created using GRADE profiler version 3.6.1 to rate the quality of all outcomes.

Measures of treatment effect

Mean differences (MDs) with 95% confidence intervals (CIs) and standardized mean differences (SMDs) with 95% confidence intervals (CIs) will be used for continuous outcome data with the same unit and different units, respectively. The relative risks (RRs) with 95% CIs will be used for dichotomous outcome data. Heterogeneity analyses will be performed by

For peer review only - http://bmjopen.bmj.com/site/about/guidelines.xhtml

Review Manager version 5.4 (Rev Man, Cochrane Collaboration, Oxford, UK). Statistical heterogeneity will be assessed by the standard χ^2 test (α=0.1) and I^2 test. If $p \geq 0.1$ and if $I^2 \leq 50\%$, the fixed-effects model will be used. If $p < 0.1$ or $I^2 > 50\%$, the random-effects model will be applied. [40] A P value < 0.05 will be considered statistically significant.

Trial Sequential Analysis

The required information size (RIS) will be calculated to correct the risks of random errors by trial sequential analysis (TSA) using the TSA program version 0.9.5.10 Beta (Copenhagen Trial Unit, Copenhagen, Denmark). [48-50] TSA program version is available at http://www.ctu.dk/tsa. [51] Each outcome will be detected by the RIS, the cumulative Z-curve and the TSA monitoring boundaries. [52 53]

To calculate the RIS, the risk of type I error will be maintained at 5% with a power of 90% in both continuous and dichotomous outcomes.[54] For continuous outcomes, a mean difference of the observed SD/2 will be used to calculate the RIS. For dichotomous outcomes, the proportion or percentage from the control group, a relative risk reduction (RRR) of 20% or -20% will be used to calculate the RIS. When there is limited evidence available about the intervention under investigation, we will estimate a clinically relevant intervention effect by using clinical experience and evidence from related areas. [53-55]

Subgroup analysis

For peer review only - http://bmjopen.bmj.com/site/about/guidelines.xhtml

The results will be comprehensively interpreted through an analysis of subgroups or subsets as much as possible. If sufficient trials are available, data from different participants' ages, and different locations of DNAs will be analyzed independently.

► Different participants' ages (infants; children; adults; aged).

► Different locations of DNAs (parapharyngeal space, submandibular space, masseter space, retropharyngeal space, sublingual space, prevertebral space, and carotid space).

The interaction p value will be used to test the statistically significant subgroup difference. If a significant difference between subgroups exists (testing for interaction $p<0.05$), the results for individual subgroups will be reported separately. [40]

Assessment of publication biases

Visual judgment of the funnel plot asymmetry and Egger's regression test will be performed to estimate the potential publication bias, while more than 10 original studies involved the same outcome measure. [56 57] Analyses of reporting bias will be performed by Stata/MP 16.0 (Stata Corp, College Station, TX, USA). The effect sizes of each included study will be normally symmetrically distributed around the center of a funnel plot in the absence of publication bias.[56] In the case of publication bias, trim and fill analysis will be performed to estimate the number of missing studies that might exist and amend the bias. [58] A level of $P < 0.05$ will be

For peer review only - http://bmjopen.bmj.com/site/about/guidelines.xhtml

considered statistically significant and indicated potential publication bias.

Grading the quality of evidence

The quality of evidence for each outcome will be assessed using the Grading of Recommendations Assessment, Development and Evaluation (GRADE) criteria. [59] The quality of effect estimates will be classified as high, moderate, low or very low depending on the risk of bias, consistency, directness, precision and publication bias.[59] Data from randomized controlled trials will be classified as high-quality evidence according to GRADE. However, it can be degraded according to risk of bias, imprecision, inconsistency, indirectness or publication bias.

ETHICS AND DISSEMINATION

Ethical approval was not required for this systematic review protocol. The findings will be disseminated through peer-reviewed publications.

Timelines

Formal screening of search results will begin in September 2023. Data extraction will begin in December 2023. The project will be completed in March 2024.

Author Contributions

Z-JQ and DL conceived the idea for this systematic review. All authors (NW, DL, CG, Z-YB, Y-XB, LB, Z-WY and Z-JQ) developed the methodology for the systematic review. The manuscript was drafted by Z-JQ and CG, and revised by all authors. NW and DL will screen potential

For peer review only - http://bmjopen.bmj.com/site/about/guidelines.xhtml

studies and extract the data. Z-YB and Z-WY will undertake risk of bias assessment and assess the evidence quality. NW and Z-JQ will conduct the data synthesis. All authors contributed to the research and agreed to be responsible for all aspects of the work.

Funding

None.

Competing interests

None declared.

Data availability statement

Not applicable for this protocol.

Patient consent for publication

No patient was involved.

Provenance and peer review

Not commissioned; externally peer reviewed.

REFERENCES

1 Larawin V, Naipao J, Dubey SP. Head and neck space infections. *Otolaryngol Head Neck Surg* 2006; 135:889-93.

2 Santos Gorjón P, Blanco Pérez P, Morales Martín AC, *et al*. Deep neck infection. Review of 286 cases. *Acta Otorrinolaringol Esp* 2012; 63:31-41.

3 Almutairi DM, Alqahtani RM, Alshareef N, *et al*. Deep Neck Space Infections: A Retrospective Study of 183 Cases at a Tertiary Hospital. *Cureus* 2020;12: e6841.

4 Stalfors J, Adielsson A, Ebenfelt A, *et al*. Deep neck space infections remain a surgical challenge. A study of 72 patients. *Acta Otolaryngol* 2004; 124:1191-6.

For peer review only - http://bmjopen.bmj.com/site/about/guidelines.xhtml

5 Desa C, Tiwari M, Pednekar S, *et al*. Etiology and Complications of Deep Neck Space Infections: A Hospital Based Retrospective Study. *Indian J Otolaryngol Head Neck Surg* 2023; 75:697-706.

6 Wang LF, Kuo WR, Tsai SM, *et al*. Characterizations of life threatening deep cervical space infections: A review of one hundred ninety-six cases. *Am J Otolaryngol* 2003; 24:111-7.

7 Nasir SB, Shuaibu IY, Labaran SA, *et al*. Management of Deep Neck Space Infections in a Tertiary Center in North West Nigeria. *Niger J Surg* 2019; 25:183-187.

8 Velhonoja J, Lääveri M, Soukka T, *et al*. Deep neck space infections: an upward trend and changing characteristics. *Eur Arch Otorhinolaryngol* 2020; 277:863-872.

9 Dalla Torre D, Brunold S, Kisielewsky I, *et al*. Life-threatening complications of deep neck space infections. *Wien Klin Wochenschr* 2013; 125:680-6.

10 Priyamvada S, Motwani G. A Study on Deep Neck Space Infections. *Indian J Otolaryngol Head Neck Surg* 2019; 71:912-917.

11 Lee YQ, Kanagalingam J. Deep neck abscesses: the Singapore experience. *Eur Arch Otorhinolaryngol* 2011; 268:609-14.

12 Potter JK, Herford AS, Ellis E 3rd. Tracheotomy versus endotracheal intubation for airway management in deep neck space infections. *J Oral Maxillofac Surg* 2002; 60:349-54;

13 Bhardwaj R, Makkar S, Gupta A, *et al*. Deep Neck Space Infections: Current Trends and Intricacies of Management? *Indian J Otolaryngol Head Neck Surg*

For peer review only

For peer review only - http://bmjopen.bmj.com/site/about/guidelines.xhtml

2022; 74:2344-2349.

14 Boscolo RP, Stellin M, Muzzi E, *et al*. Deep neck space infections: A study of 365 cases highlighting recommendations for management and treatment. *Eur Arch Otorhinolaryngol* 2012; 269: 1241-49.

15 Bakir S, Tanriverdi MH, Gun R, *et al*. Deep neck space infections: A retrospective review of 173 cases. *Am J Otolaryngol* 2012; 33:56-63.

16 Daramola OO, Flanagan CE, Maisel RH, *et al*. Diagnosis and treatment of deep neck space abscesses. *Otolaryngol Head Neck Surg* 2009; 141:123-30.

17 Sheikh Z, Yu B, Heywood E, *et al*. The assessment and management of deep neck space infections in adults: A systematic review and qualitative evidence synthesis. *Clin Otolaryngol* 2023; 48:540-562.

18 Wong DK, Brown C, Mills N, *et al*. To drain or not to drain-management of pediatric deep neck abscesses: a case-control study. *Int J Pediatr Otorhinolaryngol* 2012; 76:1810-3.

19 Carbone PN, Capra GG, Brigger MT. Antibiotic therapy for pediatric deep neck abscesses: a systematic review. *Int J Pediatr Otorhinolaryngol* 2012; 76:1647-53.

20 Joo BY, Jang AL, Lee JH, *et al*. Application of ultrasound-guided pigtail catheter drainage for abscesses in the head and neck. *Clin Otolaryngol* 2017; 42:1087-1091.

21 Baatenburg de Jong RJ, Rongen RJ, Lameris JS, *et al*. Ultrasound-guided percutaneous drainage of deep neck abscesses. *Clin Otolaryngol* 1990; 15:159-66.

22 Gaspari RJ, Sanseverino A, Gleeson T. Abscess Incision and Drainage With or Without Ultrasonography: A Randomized Controlled Trial. *Ann Emerg Med* 2019;

For peer review only

73:1-7.

23 Ochi K, Ogino S, Fukamizu K, *et al*. US-guided drainage of deep neck space abscess. *Acta Otolaryngol Suppl* 1996; 522:120-3.

24 Chang KP, Chen YL, Hao SP, *et al*. Ultrasound-guided closed drainage for abscesses of the head and neck. *Otolaryngol Head Neck Surg* 2005;132: 119-24.

25 Biron VL, Kurien G, Dziegielewski P, *et al*. Surgical vs ultrasound-guided drainage of deep neck space abscesses: a randomized controlled trial: surgical vs ultrasound drainage. *J Otolaryngol Head Neck Surg* 2013; 42:18.

26 Dabirmoghaddam P, Mohseni A, Navvabi Z, *et al*. Is ultrasonography-guided drainage a safe and effective alternative to incision and drainage for deep neck space abscesses? *J Laryngol Otol* 2017; 131:259-263.

27 Limardo A, Blanco L, Menéndez J, *et al*. Ultrasound-guided drainage vs surgical drainage of deep neck space abscesses: A randomized controlled trial. *Acta Otorrinolaringol Esp (Engl Ed)* 2022; 73:4-10.

28 Fan X, Tao S. Comparison of ultrasound-guided puncture drainage and incision drainage for deep neck abscess. *Gland Surg* 2021;10: 1431-1438.

29 Strassen U, Grimler C, Hofauer B. Ultrasound-Guided Needle Aspiration vs. Surgical Incision of Parotid Abscesses. *J Clin Med* 2022; 11:7425.

30 Delides A, Manoli E, Papadopoulos M, *et al*. Ultrasound-guided transoral drainage of a paediatric parapharyngeal abscess. *J Laryngol Otol* 2014; 128:1120-2.

31 Hagiwara Y, Araga T, Saito Y, *et al*. Utility of transoral pharyngeal ultrasonography for puncture drainage of peritonsillar abscess. *Clin Case Rep*

For peer review only - http://bmjopen.bmj.com/site/about/guidelines.xhtml

2019;7: 839-840.

32 Yeow KM, Liao CT, Hao SP. US-guided needle aspiration and catheter drainage as an alternative to open surgical drainage for uniloculated neck abscesses. *J Vasc Interv Radiol* 2001;12: 589-94.

33 Zeng DS, Chen JY, Yu D, *et al*. Application of Percutaneous Puncture in Maxillofacial and Cervical Space Infection with the Guide of B-mode Ultrasound. *Journal of Medical Research* 2019; 48:121-124.

34 Lin Y, An L, Li L. New treatment choice of head and neck cellulitis: multi-catheter drainage guided by ultrasound percutaneous puncture. *Chinese Journal of Otorhinolaryngology Head and Neck Surgery* 2017; 52:110-2.

35 Baek MY, Park KH, We JH, *et al*. Needle aspiration as therapeutic management for suppurative cervical lymphadenitis in children. *Korean J Pediatr* 2010; 53:801-4.

36 Serour F, Gorenstein A, Somekh E. Needle aspiration for suppurative cervical lymphadenitis. *Clin Pediatr (Phila)* 2002;41: 471-4.

37 Dunya G, Keefe KR, Orb Q, *et al*. Surgical vs interventional radiology drainage of neck abscesses in pediatric patients. *Int J Pediatr Otorhinolaryngol* 2023; 164:111382.

38 Lawrence R, Bateman N. Controversies in the management of deep neck space infection in children: an evidence-based review. *Clin Otolaryngol* 2017; 42:156-163.

39 Shamseer L, Moher D, Clarke M. Preferred reporting items for systematic review

For peer review only

For peer review only - http://bmjopen.bmj.com/site/about/guidelines.xhtml

and meta-analysis protocols (PRISMA-P) 2015: elaboration and explanation. *BMJ* 2015; 350: g7647.

40 Higgins JPT, Thomas J, Chandler J, *et al*. Cochrane Handbook for Systematic Reviews of Interventions version 6.2 (updated February 2021). Cochrane, 2021. Available: *www.training.cochrane.org/handbook.*

41 Page MJ, McKenzie JE, Bossuyt PM, *et al*. The PRISMA 2020 statement: an updated guideline for reporting systematic reviews. *BMJ* 2021; 372: n71.

42 Gheibi S, Mahmoodzadeh A, Kashfi K, Jeddi S, Ghasemi A. Data Extraction from Graphs Using Adobe Photoshop: Applications for Meta-Analyses. *Int J Endocrinol Metab* 2019;17: e95216.

43 Luo D, Wan X, Liu J, Tong T. Optimally estimating the sample mean from the sample size, median, mid-range, and/or mid-quartile range. *Stat Methods Med Res 2018*; 27:1785-1805.

44 Wan X, Wang W, Liu J, Tong T. Estimating the sample mean and standard deviation from the sample size, median, range and/or interquartile range. *BMC Med Res Methodol* 2014; 14:135.

45 Sterne JAC, Savović J, Page MJ, *et al*. RoB 2: a revised tool for assessing risk of bias in randomised trials. *BMJ* 2019; 366: l4898.

46 Savović J, Turner RM, Mawdsley D, *et al*. Association between Risk of-Bias assessments and results of randomized trials in Cochrane reviews: the ROBES Meta-Epidemiologic study. *Am J Epidemiol* 2018;187: 1113-22.

47 Guyatt G, Oxman AD, Akl EA, *et al.* Grade guidelines: 1. Introduction-GRADE evidence profiles and summary of findings tables. *J Clin Epidemiol* 2011; 64:383-

For peer review only - http://bmjopen.bmj.com/site/about/guidelines.xhtml

94.

48 Wetterslev J, Thorlund K, Brok J, *et al*. Estimating required information size by quantifying diversity in random-effects model meta-analyses. *BMC Med Res Methodol* 2009; 9:86.

49 Wetterslev J, Jakobsen JC, Gluud C. Trial sequential analysis in systematic reviews with meta-analysis. *BMC Med Res Methodol* 2017; 17:39.

50 Wetterslev J, Thorlund K, Brok J, *et al*. Trial sequential analysis may establish when firm evidence is reached in cumulative meta-analysis. *J Clin Epidemiol* 2008; 61: 64-75.

51 Thorlund K, Engstrøm J, Wetterslev J, *et al.* User manual for trial sequential analysis (TSA) Copenhagen trial unit, centre for clinical intervention research, Denmark; 2011.Available: http://www.ctu.dk/tsa [Accessed 2023].

52 Imberger G, Thorlund K, Gluud C, *et al*. False-Positive findings in Cochrane meta-analyses with and without application of trial sequential analysis: an empirical review. *BMJ Open* 2016;6: e011890.

53 Brok J, Thorlund K, Gluud C, *et al*. Trial sequential analysis reveals insufficient information size and potentially false positive results in many meta-analyses. *J Clin Epidemiol* 2008; 61:763-9.

54 Norman GR, Sloan JA, Wyrwich KW. Interpretation of changes in health-related quality of life: the remarkable universality of half a standard deviation. *Med Care* 2003; 41:582-92.

55 Miladinovic B, Mhaskar R, Hozo I, *et al*. Optimal information size in trial

For peer review only

For peer review only - http://bmjopen.bmj.com/site/about/guidelines.xhtml

sequential analysis of time-to-event outcomes reveals potentially inconclusive results because of the risk of random error. *J Clin Epidemiol* 2013; 66:654-9.

56 Egger M, Davey Smith G, Schneider M, *et al*. Bias in meta-analysis detected by a simple, graphical test. *BMJ* 1997;315: 629-34.

57 Sterne JA, Gavaghan D, Egger M. Publication and related bias in meta-analysis: power of statistical tests and prevalence in the literature. *J Clin Epidemiol* 2000; 53:1119-29.

58 Mavridis D, Salanti G. How to assess publication bias: funnel plot, trim-and-fill method and selection models. *Evid Based Ment Health* 2014; 17:30.

59 Guyatt G, Oxman AD, Akl EA, *et al.* Grade guidelines: 1. Introduction-GRADE evidence profiles and summary of findings tables. *J Clin Epidemiol* 2011; 64:383-94.

Figure Legends

Figure 1. The PRISMA flow diagram. PRISMA, Preferred Reporting Items for Systematic Reviews and Meta-analysis. *Consider, if feasible to do so, reporting the number of records identified from each database or register searched (rather than the total number across all databases/registers). **If automation tools were used, indicate how many records were excluded by a human and how many were excluded by automation tools.

For peer review only

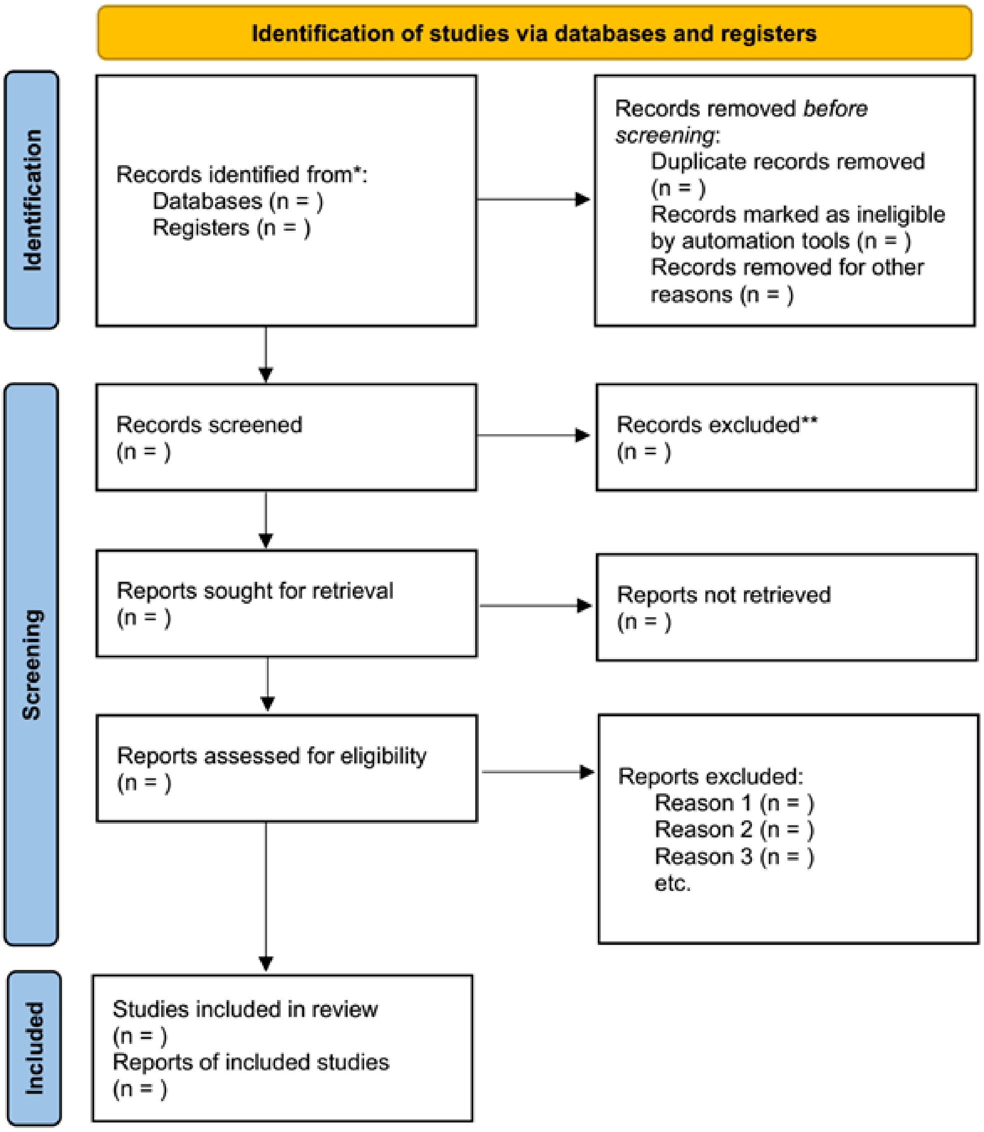

The PRISMA flow diagram

157x180mm (96 x 96 DPI)

Supplementary Appendix file 1: Search strategy

Search strategy of PubMed as follows:

#1 "deep"[All Fields] AND ("neck"[MeSH Terms] OR "neck"[All Fields]) AND ("space"[All Fields] OR "space s"[All Fields] OR "spaces"[All Fields])) OR "deep neck space"[Title/Abstract] "parapharyngeal space"[Title/Abstract] OR "submandibular space"[Title/Abstract] OR "masseter space"[Title/Abstract] OR "retropharyngeal space "[Title/Abstract] OR "sublingual space "[Title/Abstract] OR "prevertebral space "[Title/Abstract] OR "carotid space"[Title/Abstract]

#2 "infections"[MeSH Terms] OR "infection" [Text Word] OR "infections" [Text Word] OR "infections"[Title/Abstract] OR "infection"[Title/Abstract]

#3 "abscess"[MeSH Terms] OR "abscess " [Text Word] OR "abscesses" [Text Word] OR "abscess " [Title/Abstract] OR "abscesses "[Title/Abstract]

#4 #2 OR #3

#5 #1 and #4

#6 "Ultrasonography, Interventional"[Mesh] OR "Ultrasonics"[Mesh] OR "Ultrasonography"[Mesh] OR "ultrasound-guided "[Title/Abstract]

#7 "Surgical Procedures, Operative"[Mesh] OR ("Surgical"[All Fields] AND "procedures"[All Fields] AND "operative"[All Fields]) OR "operative surgical procedures"[All Fields] OR "Surgical"[All Fields] OR "surgically"[All Fields] OR "surgicals"[All Fields]) AND "Surgical"[Title/Abstract]

#8 "Randomized Controlled Trial" [Publication Type] OR "Randomized Controlled Trials as Topic"[Mesh] OR "Randomized Controlled Trial, Veterinary" [Publication Type] OR "Controlled Clinical Trial" [Publication Type] OR “randomized” [Title/Abstract] OR “randomized” [Title/Abstract] OR “Placebo” [Title/Abstract] OR “randomly” [Title/Abstract] OR “Clinical trial” [Title]

#9 #5 and #6 and #7 and #8

Search strategy of Cochrane library as follows:

#1 (deep neck space or parapharyngeal space or submandibular space or masseter space or retropharyngeal space or sublingual space or prevertebral space or carotid space): ti,ab,kw

#2 MeSH descriptor: Abscess Explode all trees

#3 (abscess): ti,ab,kw

For peer review only - http://bmjopen.bmj.com/site/about/guidelines.xhtml

#4 #2or #3

#5 MeSH descriptor: Infections Explode all trees

#6 (infections): ti,ab,kw

#7 #5or #6

#8 #4or #7

#9 #1 and #8

#10 MeSH descriptor: Ultrasonography Explode all trees

#11 (ultrasound-guided): ti,ab,kw

#12 #10 or #11

#13MeSH descriptor: Surgical Procedures, Operative Explode all trees

#14 (surgical): ti,ab,kw

#15 #13or #14

#16 (controlled clinical trial):pt or (randomized controlled trial):pt or (random*): ti,ab,kw or (Clinical trial):ti,ab,kw

#17 #9 and #12 and #15 and #16

Search strategy of Web of Science as follows:

#1 TS= (deep neck space or parapharyngeal space or submandibular space or masseter space or retropharyngeal space or sublingual space or prevertebral space or carotid space)

#2 TS= (infection)

#3 TS= (abscess)

#4 #2 or #3

#5 #1and #4

#6 TS= (surgical)

#7 TS= (ultrasound)

#8 #6 and #7

#9 TS= (random* or Clinical trial)

#10 #5 and #8 and #9

Search strategy for Ovid Medline as follows:

#1(deep neck space or parapharyngeal space or submandibular space or masseter space or retropharyngeal space or sublingual space or prevertebral space or carotid space).mp.

For peer review only

#2 exp infections/

#3 (infections).mp.

#4 #2 or # 3

#5 exp abscess /

#6 (abscess).mp.

#7 #5 or #6

#8 #4 or #7

#9 #1 and #8

#10 exp Surgical/

#11 (Surgical).mp.

#12 #10 or #11

#13 exp ultrasound/

#14 (ultrasound).mp.

#15 #13 or #14

#16 #12 and #15

#17 randomized controlled trial.pt.

#18 controlled clinical trial.pt.

#19 randomized.ab.

#20 placebo.ab.

#21 clinical trials as topic.sh.

#22 randomly.ab.

#23 trial.ti.

#24 #17 or #18 or #19 or #20 or #21 or #22 or #23

#25 #9 and #16 and #24

Search strategy for Embase as follows:

#1 (deep neck space or parapharyngeal space or submandibular space or masseter space or retropharyngeal space or sublingual space or prevertebral space or carotid space) .mp.

#2 exp Abscess/

#3 (abscess).mp.

#4 #2 or # 3

For peer review only

#5 exp infections /

#6 (infections).mp.

#7 #5 or # 6

#8 #4 or # 7

#9 #1 and #8

#10 exp ultrasonography /

#11 (ultrasound-guided).mp.

#12 #10 or # 11

#13 #9 and #12

#14 exp Surgical Procedures /

#15 (Surgical).mp.

#16 #14 or # 15

#17 exp randomized controlled trial/

#18 (random*).mp.

#19 (placebo*).mp.

#20 Clinical trial.mp.

#21 clinical trials as topic.sh.

#22 #17 or #18 or #19 or #20 or #21

#23 #13 and #16 and #22

WHO ICTRP Trial registry

http://apps.who.int/trialsearch (WHO ICTRP register) will be searched via the advanced search page. Search terms were: (deep neck space OR parapharyngeal space OR submandibular space OR masseter space OR retropharyngeal space OR sublingual space OR prevertebral space OR carotid space) AND (abscess OR infections) AND (ultrasound).

Clinicaltrials.gov search strategy

http://clinicaltrials.gov (NIH register) will be searched via advanced search page. Search terms were: Condition or disease: (deep neck space OR parapharyngeal space OR submandibular space OR masseter space OR retropharyngeal space OR sublingual space OR prevertebral space OR carotid space) AND (abscess OR infections);

Study type: Interventional Study (clinical trial);

For peer review only

Intervention/treatment: (ultrasound-guided);

Chinese Clinical Trial Registry

https://www.chictr.org.cn/index.html (Chinese Clinical Trial Registry, ChiCTR) will be searched via Subject of Registration. Search terms were: 颈部脓肿；下颌脓肿；扁桃体周围脓肿；咽旁间隙脓肿；颌下间隙脓肿；咬肌间隙脓肿；咽后间隙脓肿；舌下间隙脓肿；椎前间隙脓肿；颈动脉间隙脓肿；颈部感染；下颌感染；扁桃体周围感染；咽旁间隙感染；颌下间隙感染；咬肌间隙感染；咽后间隙感染；舌下间隙感染；椎前间隙感染；颈动脉间隙感染；

Chinese database

China National Knowledge Infrastructure (CNKI) search strategy

(颈部[全部字段]or 下颌[全部字段]or 扁桃[全部字段]or 咽旁[全部字段]or 颌下[全部字段]or 咬肌[全部字段]or 咽后[全部字段]or 舌下[全部字段]or 椎前[全部字段]or 颈动脉 [全部字段]) and (脓肿[全部字段] or 感染[全部字段]) and (超声[全部字段] or B 超[全部字段]) and (随机[全部字段] or 对照[全部字段])

Chinese BioMedical Literature (CBM)

(颈部[全部字段]or 下颌[全部字段]or 扁桃[全部字段]or 咽旁[全部字段]or 颌下[全部字段]or 咬肌[全部字段]or 咽后[全部字段]or 舌下[全部字段]or 椎前[全部字段]or 颈动脉 [全部字段]) and (脓肿[全部字段] or 感染[全部字段]) and (超声[全部字段] or B 超[全部字段]) and (随机[全部字段] or 对照[全部字段])

VIP database

关键词=(颈部 or 下颌 or 扁桃 or 咽旁 or 颌下 or 咬肌 or 咽后 or 舌下 or 椎前 or 颈动脉) AND 关键词= (脓肿 or 感染) AND 关键词= (超声 or B 超) AND 关键词= (随机 or 对照)

Wan fang database.

(颈部[全部字段]or 下颌[全部字段]or 扁桃[全部字段]or 咽旁[全部字段]or 颌下[全部字段]or 咬肌[全部字段]or 咽后[全部字段]or 舌下[全部字段]or 椎前[全部字段]or 颈动脉 [全部字段]) and (脓肿[全部字段] or 感染[全部字段]) and (超声[全部字段] or B 超[全部字段]) and (随机[全部字段] or 对照[全部字段])

For peer review only

Supplementary Appendix file 2 : Assessment of risk of bias

Random sequence generation

- ***Low risk:*** If sequence generation was achieved using computer random number generator or a random number table. Drawing lots, tossing a coin, shuffling cards, and throwing dice were also considered adequate if performed by an independent adjudicator.
- ***Unclear risk:*** If the method of randomisation was not specified, but the trial was still presented as being randomised.
- ***High risk:*** If the allocation sequence is not randomised or only quasi-randomised. These trials will be excluded.

Allocation concealment

- ***Low risk:*** If the allocation of patients was performed by a central independent unit, onsite locked computer or identical-looking numbered sealed envelopes.
- ***Uncertain risk:*** If the trial was classified as randomised but the allocation concealment process was not described.
- ***High risk:*** If the allocation sequence was familiar to the investigators who assigned participants.

Blinding of participants and treatment providers

- ***Low risk:*** If the participants and the treatment providers were blinded to intervention allocation and this was described.
- ***Uncertain risk:*** If the procedure of blinding was insufficiently described.
- ***High risk:*** If blinding of participants and the treatment providers was not performed.

Blinding of outcome assessment

- ***Low risk of bias:*** If it was mentioned that outcome assessors were blinded and this was described.
- ***Uncertain risk of bias:*** If it was not mentioned if the outcome assessors in the trial were blinded or the extent of blinding was insufficiently described.
- ***High risk of bias:*** If no blinding or incomplete blinding of outcome assessors was performed.

Incomplete outcome data

- ***Low risk of bias:*** If missing data were unlikely to make treatment effects depart from plausible

For peer review only

values. This could be either (1) there were no drop-outs or withdrawals for all outcomes, or (2) the numbers and reasons for the withdrawals and drop-outs for all outcomes were clearly stated and could be described as being similar to both groups. Generally, the trial is judged as at a low risk of bias due to incomplete outcome data if drop-outs are less than 5%. However, the 5% cut-off is not definitive.

- ***Uncertain risk of bias:*** If there was insufficient information to assess whether missing data were likely to induce bias on the results.
- ***High risk of bias:*** If the results were likely to be biased due to missing data either because the pattern of drop-outs could be described as being different in the two intervention groups or the trial used improper methods in dealing with the missing data (e.g. last observation carried forward).

Selective outcome reporting

- ***Low risk of bias:*** If a protocol was published before or at the time the trial was begun and the outcomes specified in the protocol were reported on. If there is no protocol or the protocol was published after the trial has begun, reporting of serious adverse events will grant the trial a grade of low risk of bias.
- ***Uncertain risk of bias:*** If no protocol was published and the outcome of serious adverse events were not reported on.
- ***High risk of bias:*** If the outcomes in the protocol were not reported on.

Other risks of bias

- ***Low risk of bias:*** If the trial appears to be free of other components that could put it at risk of bias.
- ***Unclear risk of bias:*** If the trial may or may not be free of other components that could put it at risk of bias.
- ***High risk of bias:*** If there are other factors in the trial that could put it at risk of bias (including, Design-specific risk of bias, stopped early due to some data-dependent process including a formal-stopping rule, baseline imbalance, claimed fraudulent, blocked randomization in unblinded trials, differential diagnostic activity, contamination, inappropriate measurement instrument for outcomes, deviation from the study protocol unrelated to the clinical practice, authors conducted trials on the same topic, academic bias, for-profit bias, inappropriate

For peer review only

financial conflict of interest).

Overall risk of bias

- ***Low risk of bias:*** The trial will be classified as overall 'low risk of bias' only if all of the bias domains described in the above paragraphs are classified as 'low risk of bias'.
- ***High risk of bias:*** The trial will be classified as 'high risk of bias' if any of the bias risk domains described in the above are classified as 'unclear' or 'high risk of bias'.
- We will assess the domains 'blinding of outcome assessment', 'incomplete outcome data', and 'selective out- come reporting' for each outcome result. Thus, we can assess the bias risk for each outcome assessed in addition to each trial. Our primary conclusions will be based on the results of our primary outcome results with overall low risk of bias. Both our primary and secondary conclusions will be presented in the summary of findings tables.

Criteria classification

- If all risk of bias domains were scored as having a low risk of bias, the trial was defined as having a low overall risk of bias.
- If one or more of the bias domains were scored as unclear or high risk of bias, the trial was defined as having a high overall risk of bias.
- Trials with a low risk of bias in all domains (including sequence generation, allocation concealment, blinding, incomplete data, selective outcome reporting, and other risks of bias) will be classified as having a low overall risk of bias.
- Trials with one or more of these domains scored as unclear or high risk of bias will be defined as having a high overall risk of bias.

For peer review only

PRISMA-P checklist

Table PRISMA-P (preferred reporting items for systematic review and meta-analysis protocols) 2015 checklist: recommended items to address in a systematic review protocol

Section and topic	Item No	Checklist item	Reported on page #
**Administrative information**			
Title:			
Identification	1a	Identify the report as a protocol of a systematic review	1
Update	1b	If the protocol is for an update of a previous systematic review, identify as such	None
Registration	2	If registered, provide the name of the registry (such as PROSPERO) and registration number	3,7
Authors:			
Contact	3a	Provide name, institutional affiliation, e-mail address of all protocol authors; provide physical mailing address of corresponding author	1
Contributions	3b	Describe contributions of protocol authors and identify the guarantor of the review	17-18
Amendments	4	If the protocol represents an amendment of a previously completed or published protocol, identify as such and list changes; otherwise, state plan for documenting important protocol amendments	None
Support:			
Sponsor	5b	Provide name for the review funder and/or sponsor	None
Role of sponsor or funder	5c	Describe roles of funder(s), sponsor(s), and/or institution(s), if any, in developing the protocol	None
**Introduction**			
Rationale	6	Describe the rationale for the review in the context of what is already known	4-6
Objectives	7	Provide an explicit statement of the question(s) the review will address with reference to participants, interventions, comparators, and outcomes (PICO)	6
**Methods**			
Eligibility criteria	8	Specify the study characteristics (such as PICO, study design, setting, time frame) and report characteristics (such as years considered, language, publication status) to be used as criteria for eligibility for the review	7-9

Information sources	9	Describe all intended information sources (such as electronic databases, contact with study authors, trial registers or other grey literature sources) with planned dates of coverage	8-11
Search strategy	10	Present draft of search strategy to be used for at least one electronic database, including planned limits, such that it could be repeated	8-9, S1
Study records:			
Data management	11a	Describe the mechanism(s) that will be used to manage records and data throughout the review	9-11
Selection process	11b	State the process that will be used for selecting studies (such as two independent reviewers) through each phase of the review (that is, screening, eligibility and inclusion in meta-analysis)	14-15
Data collection process	11c	Describe planned method of extracting data from reports (such as piloting forms, done independently, in duplicate), any processes for obtaining and confirming data from investigators	10-11
Data items	12	List and define all variables for which data will be sought (such as PICO items, funding sources), any pre-planned data assumptions and simplifications	10-11
Outcomes and prioritization	13	List and define all outcomes for which data will be sought, including prioritization of main and additional outcomes, with rationale	8
Risk of bias in individual studies	14	Describe anticipated methods for assessing risk of bias of individual studies, including whether this will be done at the outcome or study level, or both; state how this information will be used in data synthesis	11-15
Data synthesis	15a	Describe criteria under which study data will be quantitatively synthesised	12-14
	15b	If data are appropriate for quantitative synthesis, describe planned summary measures, methods of handling data and methods of combining data from studies, including any planned exploration of consistency (such as I^2, Kendall's τ)	12-14
	15c	Describe any proposed additional analyses (such as sensitivity or subgroup analyses, meta-regression)	13-14
	15d	If quantitative synthesis is not appropriate, describe the type of summary planned	12-13
Meta-bias(es)	16	Specify any planned assessment of meta-bias(es) (such as publication bias across studies, selective reporting within studies)	12-13
Confidence in cumulative evidence	17	Describe how the strength of the body of evidence will be assessed (such as GRADE)	14-15

BMJ Open

Ultrasound-guided puncture drainage vs surgical incision drainage for deep neck space abscesses: a protocol for a systematic review with meta-analysis and trial sequential analysis

Journal:	*BMJ Open*
Manuscript ID	bmjopen-2023-077631.R2
Article Type:	Protocol
Date Submitted by the Author:	30-Nov-2023
Complete List of Authors:	Nie, Wei; Sichuan University West China Hospital, Department of Anesthesiology<br>Du, Li; Sichuan Cancer Hospital and Research Institute, Department of Anesthesiology<br>Chen, Guo; Sichuan University West China Hospital, Department of Anesthesiology<br>Zheng, Yongbo; Sichuan University West China Hospital, Department of Otorhinolaryngology, Head & Neck Surgery<br>Yang, Xibiao; West China Hospital of Sichuan University, Department of Radiology<br>Li, Bo; West China Hospital of Sichuan University, Department of Otorhinolaryngology, Head & Neck Surgery<br>Zhang, Weiyi; Sichuan University West China Hospital, Department of Anesthesiology<br>Zheng, Jianqiao; Sichuan University West China Hospital, Department of Anesthesiology
**Primary Subject Heading**:	Ear, nose and throat/otolaryngology
Secondary Subject Heading:	Ear, nose and throat/otolaryngology, Evidence based practice, Medical management
Keywords:	Ultrasound < RADIOLOGY & IMAGING, Randomized Controlled Trial, Head & neck surgery < OTOLARYNGOLOGY

SCHOLARONE™
Manuscripts

TITLE PAGE

Ultrasound-guided puncture drainage vs surgical incision drainage for deep neck space abscesses: a protocol for a systematic review with meta-analysis and trial sequential analysis

Authors

Wei Nie[1] E-mail: nieweione@163.com
Li Du[2] E-mail: huaying-duli@163.com
Guo Chen[1] E-mail: Anesthesiology_SCU@163.com
Yongbo Zheng[3] E-mail: zheng_yongbo@126.com
Xibiao Yang[4] E-mail: yang061528@163.com
Bo Li[3] E-mail: liboent@wchscu.cn
Weiyi Zhang[1] E-mail: zhangweiyi@wchscu.cn
*Jianqiao Zheng[1] E-mail: zhjq1983@163.com

[1] Department of Anesthesiology, West China Hospital, Sichuan University No. 37th, Guoxue Alley, Wuhou District, Chengdu, Sichuan, China.

[2] Department of Anesthesiology, Sichuan Cancer Hospital & Institute, Sichuan Cancer Center, School of Medicine, University of Electronic Science and Technology of China, No.55th, People's South Road, Chengdu, Sichuan, China.

[3] Department of Otorhinolaryngology, Head & Neck Surgery, West China Hospital, Sichuan University, No. 37th, Guoxue Alley, Wuhou District, Chengdu, Sichuan, China.

[4] Department of Radiology, West China Hospital, Sichuan University, No. 37th, Guoxue Alley, Wuhou District, Chengdu, Sichuan, China.

*Corresponding Author
Name: Jianqiao Zheng
E-mail: zhjq1983@163.com
Phone: +86-28-8542-3593
Address: Department of Anesthesiology, Sichuan University West China Hospital, No. 37th, Guoxue Alley, Wuhou District, Chengdu 610041, Sichuan, China.

Words: 3010

For peer review only

For peer review only - http://bmjopen.bmj.com/site/about/guidelines.xhtml

ABSTRACT

Introduction Deep neck space abscesses (DNAs) are serious surgical emergencies, associated with life-threatening complications. Surgical incision-and-drainage combined with antibiotics is the main treatment for DNAs, but drawbacks still exist. Ultrasound-guided puncture drainage is an alternative treatment for some DNAs with limited clinical evidence. Hence, the optimal drainage technique for the treatment of DNAs remains unclear. Therefore, we will perform a protocol for a systematic review and meta-analysis to identify the efficacy of ultrasound-guided puncture drainage for DNAs.

Methods and analysis PubMed, Ovid Medline, Cochrane Library, Embase, Web of Science, China National Knowledge Infrastructure, Wanfang database, VIP database and trial registry databases will be searched from inception to September 2023 to identify randomized controlled trials of patients diagnosed DNAs accepting ultrasound-guided puncture drainage. Primary outcome will be the length of hospital stay. Secondary outcomes will be the cure rate, incidence of retreatment, complications and overall cost to the healthcare system. Fixed-effects or random-effects model will be used according to the statistical heterogeneity. Mean differences or standardized mean differences with 95% confidence intervals (CIs) for continuous data and risk ratio (RR) with 95% Cis for dichotomous data. The Cochrane risk-of-bias tool 2, Grading of

For peer review only

Recommendations Assessment, Development and Evaluation (GRADE) and trial sequential analysis will be conducted to evaluate the evidence quality and control the random errors. Funnel plots and Egger's regression test will be performed to evaluate publication bias.

Ethics and dissemination Ethical approval was not required for this systematic review protocol. The results will be disseminated through peer-reviewed publications.

Keywords deep neck space, abscesses, infection, ultrasound, surgical, meta-analysis, randomized controlled trial.

PROSPERO registration number CRD42023441031

Strengths and limitations of the study

►The Preferred Reporting Items for Systematic Review and Meta-Analysis Protocols (PRISMA-P) guidelines are stringently applied to enhance the quality of reporting of this protocol.

►Trial sequential analysis will be conducted to control random errors of all outcomes.

► The main drawback is that the conclusions will depend on the size and quality of the existing literature and may be constrained by a small number of studies as well as the heterogeneity of the patients and different locations of deep neck space abscesses

For peer review only - http://bmjopen.bmj.com/site/about/guidelines.xhtml

INTRODUCTION

Deep neck space abscesses (DNAs) are serious otolaryngology-head and neck surgery emergencies, defined as bacterial infections in the potential spaces and fascial planes of the neck, which could result in significant morbidity with potential mortality. [1-4] Dental infection is the most common cause of DNAs, and the prevalence of DNAs has been reduced with the advent of broad-spectrum antibiotics. [5] However, DNAs remains serious, spreads rapidly, and is associated with life-threatening complications.[6-9]Common and potentially life-threatening complications range from 15% to 35% in patients with DNAs, [5 10 11] including airway compromise, descending mediastinitis, pericarditis, pneumonia, pleural empyema, carotid arterial erosion, meningitis, sepsis, respiratory distress, jugular vein thrombosis, disseminated intravascular coagulation, and extracranial or intracranial extension of infection. [5 8-13] Therefore, the early detection of abscesses and thorough treatment are crucial.

Traditionally, the main treatment method for DNAs is complete surgical incision-and-drainage with appropriate administration of antibiotics. [14-19] However, surgical drainage has several drawbacks. First, the risk of inadvertent injuries to neighboring vital structures (including the internal jugular vein, carotid artery and cranial nerves) is associated with devastating complications. Second, surgical trauma danger

For peer review only - http://bmjopen.bmj.com/site/about/guidelines.xhtml

is increased as the inflammatory anatomy tissue is obscure and easily bled during incision when the abscess is deep. Third, patients suffering from surgical trauma stress become frail and more sensitive to pathogenic bacteria in the environment, increasing the risk of infection-related complications. Fourth, tumor-related DNAs could cause tumor dissemination during open surgical incision-and-drainage. Fifth, surgical incision-and-drainage was performed under general anesthesia in most circumstances, which will increase the anesthesia-related risk, especially in poor medical condition DNAs patients. Finally, surgical incision scars increase the risk of incision disunion and cause aesthetic defects. Therefore, a noninvasive, effective alternative to open surgical incision drainage may be beneficial to DNAs patients.

Minimally invasive techniques, such as ultrasound-guided puncture drainage, have been used to treat abscesses of the head and neck, even some DNAs. [20-37] Ultrasound-guided puncture drainage can avoid the above drawbacks of surgical incision drainage, and provide several advantages. First, ultrasound-guided puncture drainage can accurately locate the DNAs, eliminating trauma to the neighboring vital structures, and avoiding tumor dissemination during surgical incision. Second, the drainage tube can be placed under the real-time guidance of ultrasound, which is beneficial for thoroughly draining the abscess. Third, the real-time capability of ultrasound could facilitate dynamic observation after drainage and

For peer review only - http://bmjopen.bmj.com/site/about/guidelines.xhtml

monitoring the adequacy of drainage. Finally, ultrasound-guided puncture drainage does not cause skin nonunion or aesthetic defects. Several studies have shown that ultrasound-guided puncture drainage is a more effective option than surgical incision drainage for some DNAs. [20-36] In addition, ultrasound-guided puncture drainage can reduce the length of hospital stay. [25-29] However, most of these studies were case series, reviews and retrospective studies, and prospective and randomized controlled trials are rare.[20-36] In this situation, controversies still exist in the selection of drainage technique for the treatment of DNAs. [37 38]

Therefore, it is necessary to conduct a systematic review and meta-analysis to analyze the clinical efficacy of ultrasound-guided puncture drainage for DNAs. The outcomes of this systematic review will provide evidence for better clinical decision making and possible future directions for further clinical trials.

Objectives

We are performing this systematic review protocol with meta-analysis and trial sequential analysis (TSA) of randomized clinical trials to evaluate the clinical efficacy and safety of ultrasound-guided puncture drainage for DNAs.

METHODS AND ANALYSIS

Design and registration of the review

We reported this protocol according to the Preferred Reporting Items

For peer review only - http://bmjopen.bmj.com/site/about/guidelines.xhtml

for Systematic Reviews and Meta-Analyses Protocols (PRISMA-P) guidelines and have registered the protocol with PROSPERO 2023 (registration number: CRD42023441031). [39] We will perform this systematic review and meta-analysis based on the Cochrane Handbook and report the results following the PRISMA statement. [40 41] This study is anticipated to begin searching in September 2023 and will be completed in March 2024.

Patient and public involvement statement

Patients or the public were not involved in the design, conduct, reporting, or dissemination plans of our research.

Inclusion criteria for study selection

Types of studies

Only randomized controlled trials (RCTs) involving the clinical efficacy of ultrasound-guided puncture drainage for DNAs will be included. There will be no language restrictions.

The exclusion criteria were as follows: (1) studies comparing ultrasound-guided needle puncture aspiration and ultrasound-guided catheter drainage or studies comparing ultrasound-guided needle puncture aspiration with other minimally invasive techniques (CT-guided needle puncture aspiration); (2) studies with data that could not be used for statistical analysis after data transformation, or studies with incomplete data, or original data that could not be obtained after contacting the

For peer review only - http://bmjopen.bmj.com/site/about/guidelines.xhtml

corresponding authors; and (3) duplicate publications, letters or editorials, abstracts from conferences, and reviews.

Types of participants

Participants diagnosed DNAs who accept ultrasound-guided puncture drainage will be included. There will be no limitations on participants' age, gender, ethnicity, body mass index (BMI) or American Society of Anesthesiologists (ASA) classification.

Types of interventions/controls

The intervention group will be the participants with DNAs accepting any kind of ultrasound-guided puncture drainage, while the control group will receive any kind of surgical incision drainage for DNAs.

Types of outcome measures

Primary outcomes

The primary outcome will be the length of hospital stay.

Secondary outcomes

1. The cure rate;
2. Incidence of retreatment (including secondary puncture drainage or incision drainage);
3. Incidence of complications: any kind of complications will be included. Complication criteria included drainage-related mechanical injury (vascular injury, nerve injury, airway injury, and bleeding), infectious complications (sepsis, mediastinal infection, pericarditis, pneumonia,

For peer review only - http://bmjopen.bmj.com/site/about/guidelines.xhtml

arterial infection), airway obstruction, jugular vein thrombosis, skin nonunion and other puncture complications;

4. Overall cost to the healthcare system;

Search strategy

Two reviewers (NW and DL) will independently perform the search and any disagreements will be arbitrated by a third reviewer (Z-WY). English and Chinese electronic databases will be searched from inception to September 2023 for published literature. English databases (including PubMed, Ovid Medline, Cochrane Library, Embase and Web of Science) and Chinese databases [China National Knowledge Infrastructure (CNKI), Wanfang database and VIP Database] will be searched. In addition, reference lists of each study and the trial registry database (Clinical Trials.gov, WHO International Clinical Trials Registry Platform, and Chinese Clinical Trials Registry) will also be scrutinized as to avoid missing studies, and ongoing or unpublished clinical trials.

The following search terms will be used in the search strategy: deep neck space abscesses, ultrasound, surgical, and randomized controlled trial. Related search terms will also be translated into Chinese for literature research and study identification in Chinese databases. The search strategies are listed in Supplementary Appendix file 1. To avoid missing published studies during the systematic review preparation, we will update the literature search results prior to the final publication of systematic

For peer review only - http://bmjopen.bmj.com/site/about/guidelines.xhtml

For peer review only

reviews.

Data collection and analysis

Selection of studies

Two authors (NW and DL) will independently screen the potentially eligible studies by reading titles and abstracts. All identified and relevant full-text publications will be retrieved from the database search process by screening the full text thoroughly, obviously ineligible records will be eliminated, and the reasons for exclusion will be recorded. Any disagreement on eligibility of studies will be resolved through discussion or arbitrated by a third review coauthor (Z-JQ). A fourth reviewer (Z-WY) will check out all procedures carefully prior to the final confirmation of the data extraction. Duplicate publications and companion papers of the same trial will be discussed by all review authors. The entire search process is displayed in the PRISMA flow diagram (figure 1).

Data extraction

Two review authors (NW and DL) will extract data from all included studies according to a standardized data collection form (Excel version 2013, Microsoft Inc, Washington DC, USA). The data extraction form included the publication information (the first author's name, author's country publication year), participants' demographic data (sample size; age; gender; BMI; ASA physical status classification levels; inclusion and exclusion criteria if necessary), locations of DNAs, experience of the

For peer review only - http://bmjopen.bmj.com/site/about/guidelines.xhtml

operator, detailed information on drainage techniques (type of drainage techniques: surgical incision drainage or ultrasound-guided puncture drainage; days of drainage tube retention; and retreatment of multiple punctures or incisions), microbial cultures, antibiotic treatment(s) and any kind of outcomes including primary and secondary outcomes.

Study design characteristics, including the following domains: randomization, allocation concealment, blinding (patients, treatment providers, outcome investigators), incomplete outcome data collection and statistical analysis, and outcome reporting, will be recorded. Continuous data will be recorded as the mean± SD, and dichotomous data will be the recorded as percentages or proportions. A third review author (CG) will cross-check the data to ensure precision. We will contact the corresponding author of the research via email to acquire the missing or incomplete data for analysis as much as possible. In addition, the record will be excluded from the final review if no reply is received. Numerical data in the graphs will be extracted by Adobe Photoshop if necessary. [42] In addition, mean and standard deviation will be estimated from the median, mid-range, and/or mid-quartile range as described by Luo D and Wan X. [43 44]

Quality assessment

Risk of bias in each included study will be evaluated independently by two review authors (Z-YB and Z-WY) under the guidance of the Cochrane risk-of-bias tool 2 (RoB 2.0). [45] The methodology including

For peer review only - http://bmjopen.bmj.com/site/about/guidelines.xhtml

random sequence generation, allocation concealment, blinding (blinding of participants, personnel, and outcome assessment), incomplete outcome data, selective outcome reporting, other risks of bias and overall risk of bias will be assessed. Each included study will be evaluated by the Cochrane risk of bias tool and then classified into three levels (low risk, unclear risk and high risk of bias). [40 46] Any discrepancies will be settled through discussions by all review authors or arbitration by a third reviewer (CG). The assessment of risk of bias is listed in Supplementary Appendix fie 2.

Grading of Recommendations Assessment, Development, and Evaluation (GRADE) methodology will be used to assess the quality of the evidence for each outcome. [47] The quality of effect estimates will be classified as high, moderate, low or very low depending on the risk of bias, consistency, directness, precision, publication bias, large effect, and plausible confounding. [48] After the assessment, a table concerning the GRADE evidence profile will be created using GRADE profiler version 3.6.1 to rate the quality of all outcomes.

Measures of treatment effect

Mean differences (MDs) with 95% confidence intervals (CIs) and standardized mean differences (SMDs) with 95% confidence intervals (CIs) will be used for continuous outcome data with the same unit and different units, respectively. The relative risks (RRs) with 95% CIs will be used for

For peer review only - http://bmjopen.bmj.com/site/about/guidelines.xhtml

dichotomous outcome data. Heterogeneity analyses will be performed by Review Manager version 5.4 (Rev Man, Cochrane Collaboration, Oxford, UK). Statistical heterogeneity will be assessed by the standard χ^2 test (α=0.1) and I^2 test. If $p \geq 0.1$ and if $I^2 \leq 50\%$, the fixed-effects model will be used. If $p<0.1$ or $I^2>50\%$, the random-effects model will be applied. [40] A P value <0.05 will be considered statistically significant.

Trial Sequential Analysis

The required information size (RIS) will be calculated to correct the risks of random errors by trial sequential analysis (TSA) using the TSA program version 0.9.5.10 Beta (Copenhagen Trial Unit, Copenhagen, Denmark). [48-50] TSA program version is available at http://www.ctu.dk/tsa. [51] Each outcome will be detected by the RIS, the cumulative Z-curve and the TSA monitoring boundaries. [52 53]

To calculate the RIS, the risk of type I error will be maintained at 5% with a power of 90% in both continuous and dichotomous outcomes.[54] For continuous outcomes, a mean difference of the observed SD/2 will be used to calculate the RIS. For dichotomous outcomes, the proportion or percentage from the control group, a relative risk reduction (RRR) of 20% or -20% will be used to calculate the RIS. When there is limited evidence available about the intervention under investigation, we will estimate a clinically relevant intervention effect by using clinical experience and evidence from related areas. [53-55]

For peer review only - http://bmjopen.bmj.com/site/about/guidelines.xhtml

Subgroup analysis

The results will be comprehensively interpreted through an analysis of subgroups or subsets as much as possible. If sufficient trials are available, data from different participants' ages, and different locations of DNAs will be analyzed independently.

► Different participants' ages (infants; children; adults; aged).

► Different locations of DNAs (parapharyngeal space, submandibular space, masseter space, retropharyngeal space, sublingual space, prevertebral space, and carotid space).

The interaction p value will be used to test the statistically significant subgroup difference. If a significant difference between subgroups exists (testing for interaction $p<0.05$), the results for individual subgroups will be reported separately. [40]

Assessment of publication biases

Visual judgment of the funnel plot asymmetry and Egger's regression test will be performed to estimate the potential publication bias, while more than 10 original studies involved the same outcome measure. [56 57] Analyses of reporting bias will be performed by Stata/MP 16.0 (Stata Corp, College Station, TX, USA). The effect sizes of each included study will be normally symmetrically distributed around the center of a funnel plot in the absence of publication bias.[56] In the case of publication bias, trim and fill analysis will be performed to estimate the number of missing studies

For peer review only - http://bmjopen.bmj.com/site/about/guidelines.xhtml

that might exist and amend the bias. [58] A level of $P < 0.05$ will be considered statistically significant and indicated potential publication bias.

Grading the quality of evidence

The quality of evidence for each outcome will be assessed using the Grading of Recommendations Assessment, Development and Evaluation (GRADE) criteria. [59] The quality of effect estimates will be classified as high, moderate, low or very low depending on the risk of bias, consistency, directness, precision and publication bias.[59] Data from randomized controlled trials will be classified as high-quality evidence according to GRADE. However, it can be degraded according to risk of bias, imprecision, inconsistency, indirectness or publication bias.

ETHICS AND DISSEMINATION

Ethical approval was not required for this systematic review protocol. The findings will be disseminated through peer-reviewed publications.

Timelines

Formal screening of search results will begin in September 2023. Data extraction will begin in December 2023. The project will be completed in March 2024.

Author Contributions

Z-JQ and DL conceived the idea for this systematic review. All authors (NW, DL, CG, Z-YB, Y-XB, LB, Z-WY and Z-JQ) developed the methodology for the systematic review. The manuscript was drafted by Z-

For peer review only - http://bmjopen.bmj.com/site/about/guidelines.xhtml

JQ and CG, and revised by all authors. NW and DL will screen potential studies and extract the data. Z-YB and Z-WY will undertake risk of bias assessment and assess the evidence quality. NW and Z-JQ will conduct the data synthesis. All authors contributed to the research and agreed to be responsible for all aspects of the work.

Funding

None.

Competing interests

None declared.

Data availability statement

Not applicable for this protocol.

Patient consent for publication

No patient was involved.

Provenance and peer review

Not commissioned; externally peer reviewed.

REFERENCES

1 Larawin V, Naipao J, Dubey SP. Head and neck space infections. *Otolaryngol Head Neck Surg* 2006; 135:889-93.

2 Santos Gorjón P, Blanco Pérez P, Morales Martín AC, *et al*. Deep neck infection. Review of 286 cases. *Acta Otorrinolaringol Esp* 2012; 63:31-41.

3 Almutairi DM, Alqahtani RM, Alshareef N, *et al*. Deep Neck Space Infections: A Retrospective Study of 183 Cases at a Tertiary Hospital. *Cureus* 2020;12: e6841.

4 Stalfors J, Adielsson A, Ebenfelt A, *et al*. Deep neck space infections remain a

For peer review only - http://bmjopen.bmj.com/site/about/guidelines.xhtml

surgical challenge. A study of 72 patients. *Acta Otolaryngol* 2004; 124:1191-6.

5 Desa C, Tiwari M, Pednekar S, *et al*. Etiology and Complications of Deep Neck Space Infections: A Hospital Based Retrospective Study. *Indian J Otolaryngol Head Neck Surg* 2023; 75:697-706.

6 Wang LF, Kuo WR, Tsai SM, *et al*. Characterizations of life threatening deep cervical space infections: A review of one hundred ninety-six cases. *Am J Otolaryngol* 2003; 24:111-7.

7 Nasir SB, Shuaibu IY, Labaran SA, *et al*. Management of Deep Neck Space Infections in a Tertiary Center in North West Nigeria. *Niger J Surg* 2019; 25:183-187.

8 Velhonoja J, Lääveri M, Soukka T, *et al*. Deep neck space infections: an upward trend and changing characteristics. *Eur Arch Otorhinolaryngol* 2020; 277:863-872.

9 Dalla Torre D, Brunold S, Kisielewsky I, *et al*. Life-threatening complications of deep neck space infections. *Wien Klin Wochenschr* 2013; 125:680-6.

10 Priyamvada S, Motwani G. A Study on Deep Neck Space Infections. *Indian J Otolaryngol Head Neck Surg* 2019; 71:912-917.

11 Lee YQ, Kanagalingam J. Deep neck abscesses: the Singapore experience. *Eur Arch Otorhinolaryngol* 2011; 268:609-14.

12 Potter JK, Herford AS, Ellis E 3rd. Tracheotomy versus endotracheal intubation for airway management in deep neck space infections. *J Oral Maxillofac Surg* 2002; 60:349-54;

13 Bhardwaj R, Makkar S, Gupta A, *et al*. Deep Neck Space Infections: Current

For peer review only

Trends and Intricacies of Management? *Indian J Otolaryngol Head Neck Surg* 2022; 74:2344-2349.

14 Boscolo RP, Stellin M, Muzzi E, *et al*. Deep neck space infections: A study of 365 cases highlighting recommendations for management and treatment. *Eur Arch Otorhinolaryngol* 2012; 269: 1241-49.

15 Bakir S, Tanriverdi MH, Gun R, *et al*. Deep neck space infections: A retrospective review of 173 cases. *Am J Otolaryngol* 2012; 33:56-63.

16 Daramola OO, Flanagan CE, Maisel RH, *et al*. Diagnosis and treatment of deep neck space abscesses. *Otolaryngol Head Neck Surg* 2009; 141:123-30.

17 Sheikh Z, Yu B, Heywood E, *et al*. The assessment and management of deep neck space infections in adults: A systematic review and qualitative evidence synthesis. *Clin Otolaryngol* 2023; 48:540-562.

18 Wong DK, Brown C, Mills N, *et al*. To drain or not to drain-management of pediatric deep neck abscesses: a case-control study. *Int J Pediatr Otorhinolaryngol* 2012; 76:1810-3.

19 Carbone PN, Capra GG, Brigger MT. Antibiotic therapy for pediatric deep neck abscesses: a systematic review. *Int J Pediatr Otorhinolaryngol* 2012; 76:1647-53.

20 Joo BY, Jang AL, Lee JH, *et al*. Application of ultrasound-guided pigtail catheter drainage for abscesses in the head and neck. *Clin Otolaryngol* 2017; 42:1087-1091.

21 Baatenburg de Jong RJ, Rongen RJ, Lameris JS, *et al*. Ultrasound-guided percutaneous drainage of deep neck abscesses. *Clin Otolaryngol* 1990; 15:159-66.

22 Gaspari RJ, Sanseverino A, Gleeson T. Abscess Incision and Drainage With or

For peer review only

Without Ultrasonography: A Randomized Controlled Trial. *Ann Emerg Med* 2019; 73:1-7.

23 Ochi K, Ogino S, Fukamizu K, *et al*. US-guided drainage of deep neck space abscess. *Acta Otolaryngol Suppl* 1996; 522:120-3.

24 Chang KP, Chen YL, Hao SP, *et al*. Ultrasound-guided closed drainage for abscesses of the head and neck. *Otolaryngol Head Neck Surg* 2005;132: 119-24.

25 Biron VL, Kurien G, Dziegielewski P, *et al*. Surgical vs ultrasound-guided drainage of deep neck space abscesses: a randomized controlled trial: surgical vs ultrasound drainage. *J Otolaryngol Head Neck Surg* 2013; 42:18.

26 Dabirmoghaddam P, Mohseni A, Navvabi Z, *et al*. Is ultrasonography-guided drainage a safe and effective alternative to incision and drainage for deep neck space abscesses? *J Laryngol Otol* 2017; 131:259-263.

27 Limardo A, Blanco L, Menéndez J, *et al*. Ultrasound-guided drainage vs surgical drainage of deep neck space abscesses: A randomized controlled trial. *Acta Otorrinolaringol Esp (Engl Ed)* 2022; 73:4-10.

28 Fan X, Tao S. Comparison of ultrasound-guided puncture drainage and incision drainage for deep neck abscess. *Gland Surg* 2021;10: 1431-1438.

29 Strassen U, Grimler C, Hofauer B. Ultrasound-Guided Needle Aspiration vs. Surgical Incision of Parotid Abscesses. *J Clin Med* 2022; 11:7425.

30 Delides A, Manoli E, Papadopoulos M, *et al*. Ultrasound-guided transoral drainage of a paediatric parapharyngeal abscess. *J Laryngol Otol* 2014; 128:1120-2.

31 Hagiwara Y, Araga T, Saito Y, *et al*. Utility of transoral pharyngeal

For peer review only - http://bmjopen.bmj.com/site/about/guidelines.xhtml

ultrasonography for puncture drainage of peritonsillar abscess. *Clin Case Rep* 2019;7: 839-840.

32 Yeow KM, Liao CT, Hao SP. US-guided needle aspiration and catheter drainage as an alternative to open surgical drainage for uniloculated neck abscesses. *J Vasc Interv Radiol* 2001;12: 589-94.

33 Zeng DS, Chen JY, Yu D, *et al*. Application of Percutaneous Puncture in Maxillofacial and Cervical Space Infection with the Guide of B-mode Ultrasound. *Journal of Medical Research* 2019; 48:121-124.

34 Lin Y, An L, Li L. New treatment choice of head and neck cellulitis: multi-catheter drainage guided by ultrasound percutaneous puncture. *Chinese Journal of Otorhinolaryngology Head and Neck Surgery* 2017; 52:110-2.

35 Baek MY, Park KH, We JH, *et al*. Needle aspiration as therapeutic management for suppurative cervical lymphadenitis in children. *Korean J Pediatr* 2010; 53:801-4.

36 Serour F, Gorenstein A, Somekh E. Needle aspiration for suppurative cervical lymphadenitis. *Clin Pediatr (Phila)* 2002;41: 471-4.

37 Dunya G, Keefe KR, Orb Q, *et al*. Surgical vs interventional radiology drainage of neck abscesses in pediatric patients. *Int J Pediatr Otorhinolaryngol* 2023; 164:111382.

38 Lawrence R, Bateman N. Controversies in the management of deep neck space infection in children: an evidence-based review. *Clin Otolaryngol* 2017; 42:156-163.

For peer review only

For peer review only - http://bmjopen.bmj.com/site/about/guidelines.xhtml

39 Shamseer L, Moher D, Clarke M. Preferred reporting items for systematic review and meta-analysis protocols (PRISMA-P) 2015: elaboration and explanation. *BMJ* 2015; 350: g7647.

40 Higgins JPT, Thomas J, Chandler J, *et al*. Cochrane Handbook for Systematic Reviews of Interventions version 6.2 (updated February 2021). Cochrane, 2021. Available: *www.training.cochrane.org/handbook.*

41 Page MJ, McKenzie JE, Bossuyt PM, *et al*. The PRISMA 2020 statement: an updated guideline for reporting systematic reviews. *BMJ* 2021; 372: n71.

42 Gheibi S, Mahmoodzadeh A, Kashfi K, Jeddi S, Ghasemi A. Data Extraction from Graphs Using Adobe Photoshop: Applications for Meta-Analyses. *Int J Endocrinol Metab* 2019;17: e95216.

43 Luo D, Wan X, Liu J, Tong T. Optimally estimating the sample mean from the sample size, median, mid-range, and/or mid-quartile range. *Stat Methods Med Res 2018*; 27:1785-1805.

44 Wan X, Wang W, Liu J, Tong T. Estimating the sample mean and standard deviation from the sample size, median, range and/or interquartile range. *BMC Med Res Methodol* 2014; 14:135.

45 Sterne JAC, Savović J, Page MJ, *et al*. RoB 2: a revised tool for assessing risk of bias in randomised trials. *BMJ* 2019; 366: l4898.

46 Savović J, Turner RM, Mawdsley D, *et al*. Association between Risk of-Bias assessments and results of randomized trials in Cochrane reviews: the ROBES Meta-Epidemiologic study. *Am J Epidemiol* 2018;187: 1113-22.

47 Guyatt G, Oxman AD, Akl EA, *et al.* Grade guidelines: 1. Introduction-GRADE

For peer review only - http://bmjopen.bmj.com/site/about/guidelines.xhtml

evidence profiles and summary of findings tables. *J Clin Epidemiol* 2011; 64:383-94.

48 Wetterslev J, Thorlund K, Brok J, *et al*. Estimating required information size by quantifying diversity in random-effects model meta-analyses. *BMC Med Res Methodol* 2009; 9:86.

49 Wetterslev J, Jakobsen JC, Gluud C. Trial sequential analysis in systematic reviews with meta-analysis. *BMC Med Res Methodol* 2017; 17:39.

50 Wetterslev J, Thorlund K, Brok J, *et al*. Trial sequential analysis may establish when firm evidence is reached in cumulative meta-analysis. *J Clin Epidemiol* 2008; 61: 64-75.

51 Thorlund K, Engstrøm J, Wetterslev J, *et al.* User manual for trial sequential analysis (TSA) Copenhagen trial unit, centre for clinical intervention research, Denmark; 2011.Available: http://www.ctu.dk/tsa [Accessed 2023].

52 Imberger G, Thorlund K, Gluud C, *et al*. False-Positive findings in Cochrane meta-analyses with and without application of trial sequential analysis: an empirical review. *BMJ Open* 2016;6: e011890.

53 Brok J, Thorlund K, Gluud C, *et al*. Trial sequential analysis reveals insufficient information size and potentially false positive results in many meta-analyses. *J Clin Epidemiol* 2008; 61:763-9.

54 Norman GR, Sloan JA, Wyrwich KW. Interpretation of changes in health-related quality of life: the remarkable universality of half a standard deviation. *Med Care* 2003; 41:582-92.

For peer review only

For peer review only - http://bmjopen.bmj.com/site/about/guidelines.xhtml

55 Miladinovic B, Mhaskar R, Hozo I, *et al*. Optimal information size in trial sequential analysis of time-to-event outcomes reveals potentially inconclusive results because of the risk of random error. *J Clin Epidemiol* 2013; 66:654-9.

56 Egger M, Davey Smith G, Schneider M, *et al*. Bias in meta-analysis detected by a simple, graphical test. *BMJ* 1997;315: 629-34.

57 Sterne JA, Gavaghan D, Egger M. Publication and related bias in meta-analysis: power of statistical tests and prevalence in the literature. *J Clin Epidemiol* 2000; 53:1119-29.

58 Mavridis D, Salanti G. How to assess publication bias: funnel plot, trim-and-fill method and selection models. *Evid Based Ment Health* 2014; 17:30.

59 Guyatt G, Oxman AD, Akl EA, *et al*. Grade guidelines: 1. Introduction-GRADE evidence profiles and summary of findings tables. *J Clin Epidemiol* 2011; 64:383-94.

Figure Legends

Figure 1. The PRISMA flow diagram. PRISMA, Preferred Reporting Items for Systematic Reviews and Meta-analysis. *Consider, if feasible to do so, reporting the number of records identified from each database or register searched (rather than the total number across all databases/registers). **If automation tools were used, indicate how many records were excluded by a human and how many were excluded by automation tools.

For peer review only - http://bmjopen.bmj.com/site/about/guidelines.xhtml

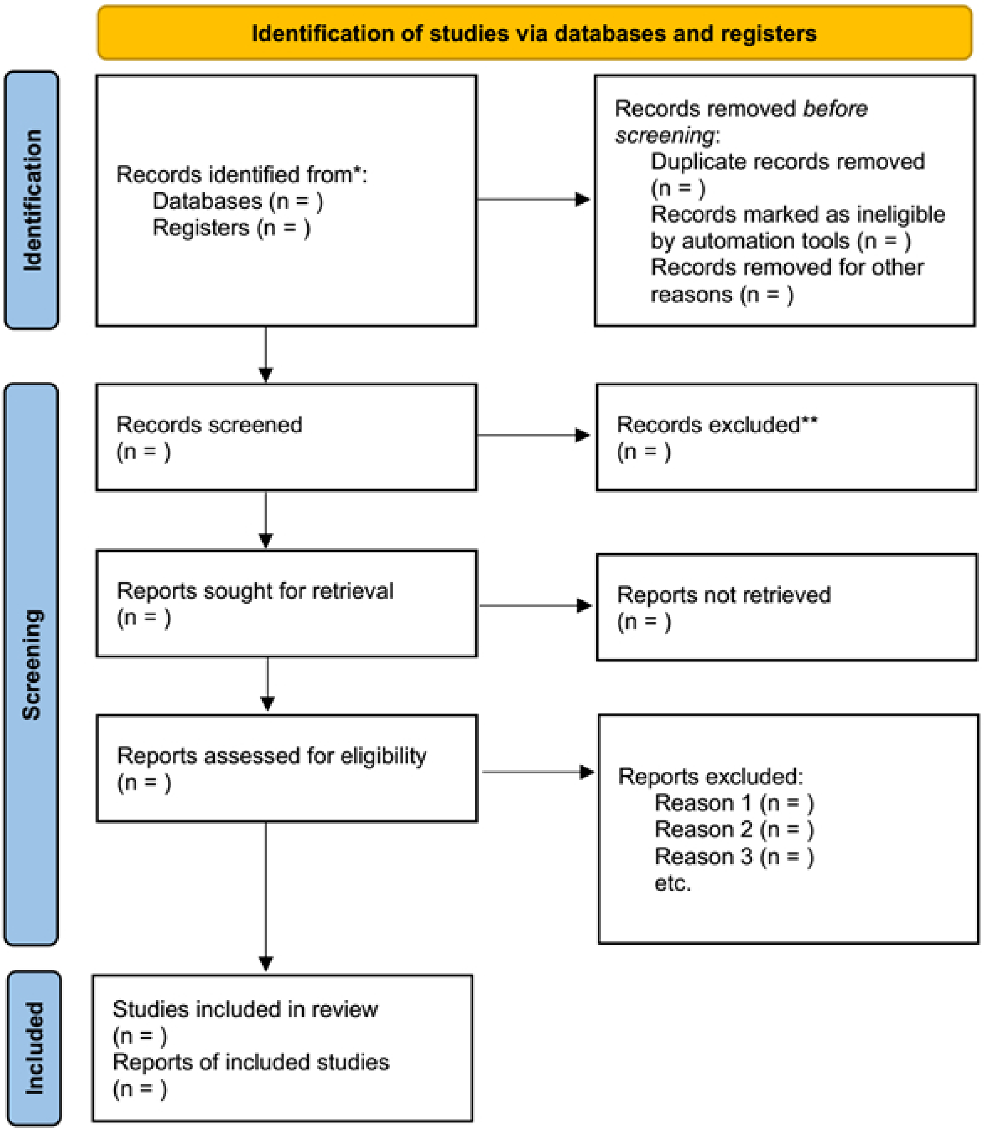

The PRISMA flow diagram

157x180mm (96 x 96 DPI)

Supplementary Appendix file 1: Search strategy

Search strategy of PubMed as follows:

#1 "deep"[All Fields] AND ("neck"[MeSH Terms] OR "neck"[All Fields]) AND ("space"[All Fields] OR "space s"[All Fields] OR "spaces"[All Fields])) OR "deep neck space"[Title/Abstract] "parapharyngeal space"[Title/Abstract] OR "submandibular space"[Title/Abstract] OR "masseter space"[Title/Abstract] OR "retropharyngeal space "[Title/Abstract] OR "sublingual space "[Title/Abstract] OR "prevertebral space "[Title/Abstract] OR "carotid space"[Title/Abstract]

#2 "infections"[MeSH Terms] OR "infection" [Text Word] OR "infections" [Text Word] OR "infections"[Title/Abstract] OR "infection"[Title/Abstract]

#3 "abscess"[MeSH Terms] OR "abscess " [Text Word] OR "abscesses" [Text Word] OR "abscess " [Title/Abstract] OR "abscesses "[Title/Abstract]

#4 #2 OR #3

#5 #1 and #4

#6 "Ultrasonography, Interventional"[Mesh] OR "Ultrasonics"[Mesh] OR "Ultrasonography"[Mesh] OR "ultrasound-guided "[Title/Abstract]

#7 "Surgical Procedures, Operative"[Mesh] OR ("Surgical"[All Fields] AND "procedures"[All Fields] AND "operative"[All Fields]) OR "operative surgical procedures"[All Fields] OR "Surgical"[All Fields] OR "surgically"[All Fields] OR "surgicals"[All Fields]) AND "Surgical"[Title/Abstract]

#8 "Randomized Controlled Trial" [Publication Type] OR "Randomized Controlled Trials as Topic"[Mesh] OR "Randomized Controlled Trial, Veterinary" [Publication Type] OR "Controlled Clinical Trial" [Publication Type] OR “randomized” [Title/Abstract] OR “randomized” [Title/Abstract] OR “Placebo” [Title/Abstract] OR “randomly” [Title/Abstract] OR “Clinical trial” [Title]

#9 #5 and #6 and #7 and #8

Search strategy of Cochrane library as follows:

#1 (deep neck space or parapharyngeal space or submandibular space or masseter space or retropharyngeal space or sublingual space or prevertebral space or carotid space): ti,ab,kw

#2 MeSH descriptor: Abscess Explode all trees

#3 (abscess): ti,ab,kw

For peer review only - http://bmjopen.bmj.com/site/about/guidelines.xhtml

#4 #2or #3

#5 MeSH descriptor: Infections Explode all trees

#6 (infections): ti,ab,kw

#7 #5or #6

#8 #4or #7

#9 #1 and #8

#10 MeSH descriptor: Ultrasonography Explode all trees

#11 (ultrasound-guided): ti,ab,kw

#12 #10 or #11

#13MeSH descriptor: Surgical Procedures, Operative Explode all trees

#14 (surgical): ti,ab,kw

#15 #13or #14

#16 (controlled clinical trial):pt or (randomized controlled trial):pt or (random*): ti,ab,kw or (Clinical trial):ti,ab,kw

#17 #9 and #12 and #15 and #16

Search strategy of Web of Science as follows:

#1 TS= (deep neck space or parapharyngeal space or submandibular space or masseter space or retropharyngeal space or sublingual space or prevertebral space or carotid space)

#2 TS= (infection)

#3 TS= (abscess)

#4 #2 or #3

#5 #1and #4

#6 TS= (surgical)

#7 TS= (ultrasound)

#8 #6 and #7

#9 TS= (random* or Clinical trial)

#10 #5 and #8 and #9

Search strategy for Ovid Medline as follows:

#1(deep neck space or parapharyngeal space or submandibular space or masseter space or retropharyngeal space or sublingual space or prevertebral space or carotid space).mp.

For peer review only

#2 exp infections/

#3 (infections).mp.

#4 #2 or # 3

#5 exp abscess /

#6 (abscess).mp.

#7 #5 or #6

#8 #4 or #7

#9 #1 and #8

#10 exp Surgical/

#11 (Surgical).mp.

#12 #10 or #11

#13 exp ultrasound/

#14 (ultrasound).mp.

#15 #13 or #14

#16 #12 and #15

#17 randomized controlled trial.pt.

#18 controlled clinical trial.pt.

#19 randomized.ab.

#20 placebo.ab.

#21 clinical trials as topic.sh.

#22 randomly.ab.

#23 trial.ti.

#24 #17 or #18 or #19 or #20 or #21 or #22 or #23

#25 #9 and #16 and #24

Search strategy for Embase as follows:

#1 (deep neck space or parapharyngeal space or submandibular space or masseter space or retropharyngeal space or sublingual space or prevertebral space or carotid space) .mp.

#2 exp Abscess/

#3 (abscess).mp.

#4 #2 or # 3

For peer review only

#5 exp infections /

#6 (infections).mp.

#7 #5 or # 6

#8 #4 or # 7

#9 #1 and #8

#10 exp ultrasonography /

#11 (ultrasound-guided).mp.

#12 #10 or # 11

#13 #9 and #12

#14 exp Surgical Procedures /

#15 (Surgical).mp.

#16 #14 or # 15

#17 exp randomized controlled trial/

#18 (random*).mp.

#19 (placebo*).mp.

#20 Clinical trial.mp.

#21 clinical trials as topic.sh.

#22 #17 or #18 or #19 or #20 or #21

#23 #13 and #16 and #22

WHO ICTRP Trial registry

http://apps.who.int/trialsearch (WHO ICTRP register) will be searched via the advanced search page. Search terms were: (deep neck space OR parapharyngeal space OR submandibular space OR masseter space OR retropharyngeal space OR sublingual space OR prevertebral space OR carotid space) AND (abscess OR infections) AND (ultrasound).

Clinicaltrials.gov search strategy

http://clinicaltrials.gov (NIH register) will be searched via advanced search page. Search terms were: Condition or disease: (deep neck space OR parapharyngeal space OR submandibular space OR masseter space OR retropharyngeal space OR sublingual space OR prevertebral space OR carotid space) AND (abscess OR infections);

Study type: Interventional Study (clinical trial);

For peer review only

Intervention/treatment: (ultrasound-guided);

Chinese Clinical Trial Registry

https://www.chictr.org.cn/index.html (Chinese Clinical Trial Registry, ChiCTR) will be searched via Subject of Registration. Search terms were: 颈部脓肿；下颌脓肿；扁桃体周围脓肿；咽旁间隙脓肿；颌下间隙脓肿；咬肌间隙脓肿；咽后间隙脓肿；舌下间隙脓肿；椎前间隙脓肿；颈动脉间隙脓肿；颈部感染；下颌感染；扁桃体周围感染；咽旁间隙感染；颌下间隙感染；咬肌间隙感染；咽后间隙感染；舌下间隙感染；椎前间隙感染；颈动脉间隙感染；

Chinese database

China National Knowledge Infrastructure (CNKI) search strategy

(颈部[全部字段]or 下颌[全部字段]or 扁桃[全部字段]or 咽旁[全部字段]or 颌下[全部字段]or 咬肌[全部字段]or 咽后[全部字段]or 舌下[全部字段]or 椎前[全部字段]or 颈动脉 [全部字段]) and (脓肿[全部字段] or 感染[全部字段]) and (超声[全部字段] or B 超[全部字段]) and (随机[全部字段] or 对照[全部字段])

Chinese BioMedical Literature (CBM)

(颈部[全部字段]or 下颌[全部字段]or 扁桃[全部字段]or 咽旁[全部字段]or 颌下[全部字段]or 咬肌[全部字段]or 咽后[全部字段]or 舌下[全部字段]or 椎前[全部字段]or 颈动脉 [全部字段]) and (脓肿[全部字段] or 感染[全部字段]) and (超声[全部字段] or B 超[全部字段]) and (随机[全部字段] or 对照[全部字段])

VIP database

关键词=(颈部 or 下颌 or 扁桃 or 咽旁 or 颌下 or 咬肌 or 咽后 or 舌下 or 椎前 or 颈动脉) AND 关键词= (脓肿 or 感染) AND 关键词= (超声 or B 超) AND 关键词= (随机 or 对照)

Wan fang database.

(颈部[全部字段]or 下颌[全部字段]or 扁桃[全部字段]or 咽旁[全部字段]or 颌下[全部字段]or 咬肌[全部字段]or 咽后[全部字段]or 舌下[全部字段]or 椎前[全部字段]or 颈动脉 [全部字段]) and (脓肿[全部字段] or 感染[全部字段]) and (超声[全部字段] or B 超[全部字段]) and (随机[全部字段] or 对照[全部字段])

For peer review only

Supplementary Appendix file 2 : Assessment of risk of bias

Random sequence generation

- ***Low risk:*** If sequence generation was achieved using computer random number generator or a random number table. Drawing lots, tossing a coin, shuffling cards, and throwing dice were also considered adequate if performed by an independent adjudicator.
- ***Unclear risk:*** If the method of randomisation was not specified, but the trial was still presented as being randomised.
- ***High risk:*** If the allocation sequence is not randomised or only quasi-randomised. These trials will be excluded.

Allocation concealment

- ***Low risk:*** If the allocation of patients was performed by a central independent unit, onsite locked computer or identical-looking numbered sealed envelopes.
- ***Uncertain risk:*** If the trial was classified as randomised but the allocation concealment process was not described.
- ***High risk:*** If the allocation sequence was familiar to the investigators who assigned participants.

Blinding of participants and treatment providers

- ***Low risk:*** If the participants and the treatment providers were blinded to intervention allocation and this was described.
- ***Uncertain risk:*** If the procedure of blinding was insufficiently described.
- ***High risk:*** If blinding of participants and the treatment providers was not performed.

Blinding of outcome assessment

- ***Low risk of bias:*** If it was mentioned that outcome assessors were blinded and this was described.
- ***Uncertain risk of bias:*** If it was not mentioned if the outcome assessors in the trial were blinded or the extent of blinding was insufficiently described.
- ***High risk of bias:*** If no blinding or incomplete blinding of outcome assessors was performed.

Incomplete outcome data

- ***Low risk of bias:*** If missing data were unlikely to make treatment effects depart from plausible

For peer review only

values. This could be either (1) there were no drop-outs or withdrawals for all outcomes, or (2) the numbers and reasons for the withdrawals and drop-outs for all outcomes were clearly stated and could be described as being similar to both groups. Generally, the trial is judged as at a low risk of bias due to incomplete outcome data if drop-outs are less than 5%. However, the 5% cut-off is not definitive.

- ***Uncertain risk of bias:*** If there was insufficient information to assess whether missing data were likely to induce bias on the results.
- ***High risk of bias:*** If the results were likely to be biased due to missing data either because the pattern of drop-outs could be described as being different in the two intervention groups or the trial used improper methods in dealing with the missing data (e.g. last observation carried forward).

Selective outcome reporting

- ***Low risk of bias:*** If a protocol was published before or at the time the trial was begun and the outcomes specified in the protocol were reported on. If there is no protocol or the protocol was published after the trial has begun, reporting of serious adverse events will grant the trial a grade of low risk of bias.
- ***Uncertain risk of bias:*** If no protocol was published and the outcome of serious adverse events were not reported on.
- ***High risk of bias:*** If the outcomes in the protocol were not reported on.

Other risks of bias

- ***Low risk of bias:*** If the trial appears to be free of other components that could put it at risk of bias.
- ***Unclear risk of bias:*** If the trial may or may not be free of other components that could put it at risk of bias.
- ***High risk of bias:*** If there are other factors in the trial that could put it at risk of bias (including, Design-specific risk of bias, stopped early due to some data-dependent process including a formal-stopping rule, baseline imbalance, claimed fraudulent, blocked randomization in unblinded trials, differential diagnostic activity, contamination, inappropriate measurement instrument for outcomes, deviation from the study protocol unrelated to the clinical practice, authors conducted trials on the same topic, academic bias, for-profit bias, inappropriate

For peer review only

financial conflict of interest).

Overall risk of bias

- ***Low risk of bias:*** The trial will be classified as overall 'low risk of bias' only if all of the bias domains described in the above paragraphs are classified as 'low risk of bias'.
- ***High risk of bias:*** The trial will be classified as 'high risk of bias' if any of the bias risk domains described in the above are classified as 'unclear' or 'high risk of bias'.
- We will assess the domains 'blinding of outcome assessment', 'incomplete outcome data', and 'selective out- come reporting' for each outcome result. Thus, we can assess the bias risk for each outcome assessed in addition to each trial. Our primary conclusions will be based on the results of our primary outcome results with overall low risk of bias. Both our primary and secondary conclusions will be presented in the summary of findings tables.

Criteria classification

- If all risk of bias domains were scored as having a low risk of bias, the trial was defined as having a low overall risk of bias.
- If one or more of the bias domains were scored as unclear or high risk of bias, the trial was defined as having a high overall risk of bias.
- Trials with a low risk of bias in all domains (including sequence generation, allocation concealment, blinding, incomplete data, selective outcome reporting, and other risks of bias) will be classified as having a low overall risk of bias.
- Trials with one or more of these domains scored as unclear or high risk of bias will be defined as having a high overall risk of bias.

For peer review only

PRISMA-P checklist

Table PRISMA-P (preferred reporting items for systematic review and meta-analysis protocols) 2015 checklist: recommended items to address in a systematic review protocol

Section and topic	Item No	Checklist item	Reported on page #
**Administrative information**			
Title:			
Identification	1a	Identify the report as a protocol of a systematic review	1
Update	1b	If the protocol is for an update of a previous systematic review, identify as such	None
Registration	2	If registered, provide the name of the registry (such as PROSPERO) and registration number	3,7
Authors:			
Contact	3a	Provide name, institutional affiliation, e-mail address of all protocol authors; provide physical mailing address of corresponding author	1
Contributions	3b	Describe contributions of protocol authors and identify the guarantor of the review	17-18
Amendments	4	If the protocol represents an amendment of a previously completed or published protocol, identify as such and list changes; otherwise, state plan for documenting important protocol amendments	None
Support:			
Sponsor	5b	Provide name for the review funder and/or sponsor	None
Role of sponsor or funder	5c	Describe roles of funder(s), sponsor(s), and/or institution(s), if any, in developing the protocol	None
**Introduction**			
Rationale	6	Describe the rationale for the review in the context of what is already known	4-6
Objectives	7	Provide an explicit statement of the question(s) the review will address with reference to participants, interventions, comparators, and outcomes (PICO)	6
**Methods**			
Eligibility criteria	8	Specify the study characteristics (such as PICO, study design, setting, time frame) and report characteristics (such as years considered, language, publication status) to be used as criteria for eligibility for the review	7-9

Information sources	9	Describe all intended information sources (such as electronic databases, contact with study authors, trial registers or other grey literature sources) with planned dates of coverage	8-11
Search strategy	10	Present draft of search strategy to be used for at least one electronic database, including planned limits, such that it could be repeated	8-9, S1
Study records:			
Data management	11a	Describe the mechanism(s) that will be used to manage records and data throughout the review	9-11
Selection process	11b	State the process that will be used for selecting studies (such as two independent reviewers) through each phase of the review (that is, screening, eligibility and inclusion in meta-analysis)	14-15
Data collection process	11c	Describe planned method of extracting data from reports (such as piloting forms, done independently, in duplicate), any processes for obtaining and confirming data from investigators	10-11
Data items	12	List and define all variables for which data will be sought (such as PICO items, funding sources), any pre-planned data assumptions and simplifications	10-11
Outcomes and prioritization	13	List and define all outcomes for which data will be sought, including prioritization of main and additional outcomes, with rationale	8
Risk of bias in individual studies	14	Describe anticipated methods for assessing risk of bias of individual studies, including whether this will be done at the outcome or study level, or both; state how this information will be used in data synthesis	11-15
Data synthesis	15a	Describe criteria under which study data will be quantitatively synthesised	12-14
	15b	If data are appropriate for quantitative synthesis, describe planned summary measures, methods of handling data and methods of combining data from studies, including any planned exploration of consistency (such as I^2, Kendall's τ)	12-14
	15c	Describe any proposed additional analyses (such as sensitivity or subgroup analyses, meta-regression)	13-14
	15d	If quantitative synthesis is not appropriate, describe the type of summary planned	12-13
Meta-bias(es)	16	Specify any planned assessment of meta-bias(es) (such as publication bias across studies, selective reporting within studies)	12-13
Confidence in cumulative evidence	17	Describe how the strength of the body of evidence will be assessed (such as GRADE)	14-15
